# Accurate *RET* Fusion Detection in Solid Tumors Using RNA Sequencing Coverage Imbalance Analysis

**DOI:** 10.3390/ijms262311300

**Published:** 2025-11-22

**Authors:** Ivan Gaziev, Anna Khristichenko, Daniil Luppov, Maria Suntsova, Ekaterina Bondarenko, Maria Reinberg, Alina Matrosova, Nadezhda Khilal, Maksim Sorokin, Marina Sekacheva, Elena Poddubskaya, Anton Buzdin, Galina Zakharova

**Affiliations:** 1 Institute for Personalized Oncology, Biomedical Science & Technology Park, FSAEI HE I.M. Sechenov First Moscow State Medical University of MOH of Russia (Sechenovskiy University), 119991 Moscow, Russia; gaziev_i_r@staff.sechenov.ru (I.G.); luppov.dv@phystech.edu (D.L.); suntsova_m_v@staff.sechenov.ru (M.S.); sorokin@oncobox.com (M.S.); sekacheva_m_i@staff.sechenov.ru (M.S.); poddubskaya_e_v@staff.sechenov.ru (E.P.); buzdin_a_a@staff.sechenov.ru (A.B.); 2 Department of Genetics and Life Sciences, Sirius University of Science and Technology, 354340 Sirius Federal Territory, Russia; anya.khristichenko@gmail.com; 3 National Medical Research Center for Endocrinology, 117292 Moscow, Russia; bondarenko.ekaterina@endocrincentr.ru (E.B.);; 4 Oncobox LLC., 119991 Moscow, Russia; 5 PathoBiology Group, European Organization for Research and Treatment of Cancer (EORTC), 1200 Brussels, Belgium

**Keywords:** fusion oncogene, receptor tyrosine kinase, Ret Proto-Oncogene (*RET*), *RET* rearrangement, RNA sequencing, tumor molecular diagnostics, clinical oncology

## Abstract

Accurate detection of oncogenic gene fusions is becoming increasingly important given the availability of highly effective targeted therapies. However, their identification in clinical practice remains challenging due to the rarity of individual events, diversity of partner genes, and variability of breakpoint locations. Conventional approaches such as immunohistochemistry (IHC) and fluorescence in situ hybridization (FISH) lack multiplexing capacity and demonstrate variable sensitivity and specificity, while direct identification of fusion transcripts in whole-transcriptome sequencing (RNA-seq) profiles provides broader applicability but limited sensitivity, as fusion junctions are frequently supported by a minimal number of reads or even no reads at all. In this study, a novel approach was employed to accurately detect clinically actionable *RET* (REarranged during Transfection) fusions. This approach entailed the measurement of the imbalance in RNA-seq read coverage of potential fusion oncogenes at their 3′ and 5′ exons. A total of 1327 experimental solid tumor RNA-seq profiles were screened, including 154 non-small cell lung cancer and 221 thyroid cancer samples. The *RET* status was validated in 78 selected cases by targeted NGS and Sanger sequencing. An analysis of the coverage imbalance was conducted, which enabled the accurate discrimination between true and false positive *RET* fusions. This approach outperformed other methods and yielded 100% sensitivity and specificity with optimized thresholds. The findings were validated using an independent cohort of 79 thyroid cancer cases, confirming the reliability of the results. Among the 18 *RET* fusion-positive samples, one was identified as an extremely rare case (*RUFY3::RET*), and two were determined to be novel fusions (*FN1::RET*, *PPP1R21::RET*). The findings of this study demonstrate that exon coverage imbalance analysis serves as a robust complement to computational RNA-seq analysis pipelines for the detection of clinically relevant *RET* fusions.

## 1. Introduction

The detection of fusion oncogenes is becoming increasingly significant for two primary reasons. Firstly, there has been a notable increase in the availability of new targeted medications. Secondly, molecular diagnostics methods are undergoing constant evolution, thereby facilitating more precise and effective detection of oncogenes [1]. While *RET* (REarranged during Transfection) fusions are comparatively uncommon across various human cancers, their identification is of paramount clinical significance. According to the findings of phase 1/2 studies [2,3,4], two selective RET inhibitors, selpercatinib and pralsetinib, received accelerated FDA approval in 2020 for patients with *RET* fusion-positive non-small cell lung cancer (NSCLC) and thyroid cancer only. However, the indication was subsequently expanded to include any locally advanced or metastatic solid tumors harboring a *RET* gene fusion. The objective response rates to selective RET inhibitors in *RET*-altered cancers are very high, reaching 64–70% [2,5], thus underscoring the importance of *RET* testing.

The *RET* proto-oncogene, which is located on chromosome 10 (10q11.2), encodes a transmembrane receptor tyrosine kinase [6]. RET is activated by ligands of the glial cell line-derived neurotrophic factor (GDNF) family through interaction with GFRα (GDNF family receptor-α) co-receptors. Ligand binding promotes RET dimerization, which subsequently results in the autophosphorylation of its intracellular tyrosine kinase domain. This process, in turn, activates a series of downstream signaling cascades, including the PI3K-AKT, RAS-MAPK, and JAK-STAT pathways [7,8,9]. These pathways regulate essential cellular processes, including proliferation, migration, differentiation, and neuronal guidance. RET is critical for renal and neuronal development and is generally expressed at high levels in neuronal tissues, the enteric nervous system, and the ureteric bud in adults.

**Figure 1:**
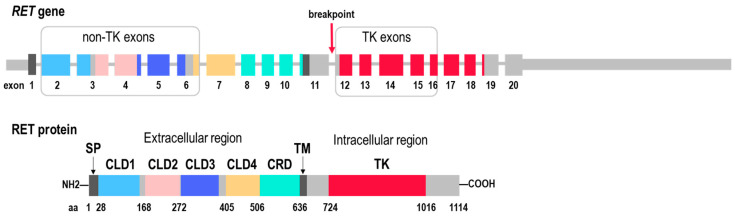
Schematic structure of the wild-type *RET* (REarranged during Transfection) gene (exons are given according to the reference transcript ENST00000355710.8) and its corresponding protein (isoform RET51). SP—signal peptide; CLD1–4—cadherin-like domains 1–4; CRD—cysteine-rich domain; TM—transmembrane domain; TK—tyrosine kinase domain. The most frequent breakpoint in *RET* rearrangements is indicated by a red arrow. Gray frames indicate groups of five exons located at the 5′ end of *RET* (“non-TK exons”) and five exons located at the 3′ end of *RET* (“TK exons”), whose expression levels were compared to assess *RET* coverage imbalance (see Section 4.2 for details).

Structurally, the extracellular region of RET comprises four cadherin-like domains (CLD1–4) and a cysteine-rich domain (CRD), whereas the intracellular region harbors a catalytically active tyrosine kinase domain (Figure 1). Alternative splicing at the 3′ end of the gene generates two major protein isoforms, RET51 (P07949-1, ENST00000355710.8) and RET9 (P07949-2, ENST00000340058.6), named according to the number of unique C-terminal amino acids. These isoforms also differ in their biological functions [10,11].

Aberrant activation of *RET* has been observed in various types of human cancer. The oncogenic fusion transcripts maintain the tyrosine kinase (TK) domain of *RET* at the 3′ end. One underlying mechanism is chromosomal rearrangement, which generates *RET* fusion transcripts and chimeric proteins. In such fusions, the partner gene frequently encodes a coiled-coil (CCD) or LIS1 homology (LisH) motif that promotes homodimerization and autophosphorylation, thereby enabling ligand-independent activation of the RET kinase [12,13,14,15]. The second mechanism of RET activation through gene fusion involves high constitutive *RET* expression driven by a ubiquitously expressed upstream partner. Subsequently, enhanced kinase activity engages downstream signaling cascades, thereby sustaining proliferative and survival programs in tumor cells.

The most frequent *RET* fusions have been observed in thyroid cancer (approximately 3% of all cases and 9–11% of the papillary subtype) and in lung adenocarcinoma (approximately 1%) [16,17]. In pediatric and adolescent patients diagnosed with papillary thyroid carcinoma, the prevalence of *RET* fusions can reach up to 30% [18].

Among the extensive list of over 100 fusion partners described for the *RET* gene [16,19,20], the most frequent 5′ partners are genes located on the same chromosome. These genes include *CCDC6* (Coiled Coil Domain Containing 6), *KIF5B* (Kinesin Family Member 5B), and *NCOA4* (Nuclear Receptor Coactivator 4). However, the predominant partner varies depending on the specific type of cancer: in the case of lung cancer, *KIF5B* accounts for 66–68% of all *RET* fusions, *CCDC6* for 18–22%, and *NCOA4* for only 2–3% [16,17]. In contrast, in thyroid cancer, the pattern is reversed, with *CCDC6* and *NCOA4* contributing 41–62% and 24–36% of rearrangements, respectively, and *KIF5B* contributing only 2–6% [16,17]. In colorectal cancer, *NCOA4* has been identified as the most prevalent partner, accounting for 63% of cases, followed by *CCDC6*, which accounts for 21% of cases, and *GPHN* (Gephyrin), accounting for 5% of cases [17]. In the vast majority of cases (87%), the *RET* breakpoint is located in intron 11, in 5% of cases in intron 10, and in other 5% in exon 11 [16,21], resulting in the loss of the extracellular and, in most cases, also the transmembrane domains (Figure 1).

A variety of methods are available for the detection of *RET* fusions. These methods include immunohistochemistry (IHC), fluorescent in situ hybridization (FISH), reverse transcription-PCR (RT-PCR), high-throughput nucleic acid hybridization technologies (e.g., NanoString), and various DNA- and RNA-based next-generation sequencing (NGS) approaches [22,23,24,25]. According to the 2021 European Society for Medical Oncology (ESMO) guidelines, the use of immunohistochemistry (IHC) is not recommended for RET fusion testing due to its relatively low (~60%) sensitivity and highly variable specificity, ranging from 40% to 85% [26,27]. Furthermore, the sensitivity of IHC is contingent upon the fusion partner, with *KIF5B* exhibiting 100% sensitivity and *NCOA4* demonstrating approximately 50% sensitivity [27].

FISH is the standard method for *RET* fusion detection, providing single-cell resolution and the capacity to discern rearrangements irrespective of the fusion partner. However, it has notable limitations, including challenges in interpretation (particularly in the context of pericentric fusions, when partners are located close to *RET*, such as *KIF5B* and *NCOA4*, or in the presence of deletions), labor intensity, lack of standardized cutoff criteria, and subjectivity in interpretation [26,27]. FISH is unable to identify the fusion partner, which has the potential to be clinically relevant for the selection of therapy, as demonstrated in the case of *KIF5B::RET*, which has been associated with poor response to multi-kinase inhibitors [28,29,30]. Furthermore, positive FISH results are not always confirmed at the transcript level [27,31,32].

Alternatively, molecular genetic methods are increasingly being utilized in clinical practice for fusion screening purposes [33]. In light of the considerable diversity inherent in potential fusion partners, methodologies that facilitate partner-agnostic fusion detection assume particular significance. These primarily include high-throughput sequencing approaches, such as whole-genome sequencing (WGS), whole-transcriptome sequencing (WTS), and targeted DNA and/or RNA sequencing [34]. Notably, among NGS-based methods, RNA-based NGS is the only approach capable of assessing the functionality of the rearrangement, encompassing the presence of transcripts, the preservation of reading frames, and the evaluation of kinase domain integrity [1,25,27].

Targeted sequencing is a method that typically involves the use of hybridization probe panels or amplification-based enrichment of target genes. In the latter case, anchored PCR is employed, which uses a combination of a gene-specific primer and an adapter-ligated primer to amplify target regions [35]. This approach facilitates the amplification of the fusion junction, even in cases where the precise fusion partner is not known. For instance, this strategy has been implemented in the commercial Archer FusionPlex platform [36] and in the ATOM-Seq method [37].

An alternative methodology for identifying 3′ fusions of druggable tyrosine kinases with unknown partners or uncommon breakpoints involves the analysis of 5′/3′ coverage imbalance [25]. This approach is based on the premise that, in the presence of a fusion, the 3′ end of the target gene is preserved in the transcript, while the 5′ end may be lost or truncated. This results in increased expression of the 3′ end relative to the 5′ end, which can be detected through various methods. For instance, disparities in expression levels can be evaluated by comparing threshold cycles in RT-qPCR for primer pairs designed at the 5′ and 3′ ends of the gene [38,39]. In amplicon-based next-generation sequencing (NGS) panels, in addition to primers targeting known fusions, primers can also be included for the 3′ and 5′ ends of genes, as in the Oncomine and AmpliSeq panels [40,41]. In the context of NanoString technology, this objective is accomplished through the utilization of fluorescence-labeled probes that are designed to target a 5′ region situated upstream of the kinase domain exons and a 3′ region [41,42]. As is the case with PCR-based methods, the NanoString approach necessitates prior selection and validation of probes for all target genes. Consequently, the analysis of 5′/3′ expression imbalance is frequently employed as a complementary approach to detect predefined fusions when partner-agnostic fusion detection is not viable.

However, the same phenomenon can also be utilized in another group of methods involving RNA-based sequencing [25]. In sum, RNA-seq provides a comprehensive means of characterizing individual tumor-specific expression profiles. The knowledge of tumor-activated drug target genes and molecular pathways can guide personalized prescription of targeted therapeutics in clinical oncology. This approach offers approximately a 10-month benefit per treatment line in progression-free survival for patients with advanced solid cancers, as demonstrated in a recent prospective clinical study [43]. Additionally, bulk tumor RNA-seq profiles can be used to detect chimeric sequencing reads indicative of fusion oncogenes [44]. However, in RNA-seq profiles, fusion events are often supported by only a few junction reads (typically 1–5) [33], which complicates the distinction between true fusions and random chimeric artifacts introduced during library preparation [25].We have demonstrated that incorporating RNA-seq coverage 5′/3′ expression asymmetry analysis in addition to fusion-read detection significantly enhances the sensitivity of *ALK* fusion identification, while maintaining specificity near 1 [34].

Nevertheless, the efficacy of 5′/3′ imbalance analysis is contingent upon numerous factors, including baseline expression levels of 5′- and 3′-fusion partner genes, splicing and isoform patterns, alternative gene transcripts, and overlapping antisense RNAs [34]. Therefore, for each type of fusion oncogene, specific investigation is required to explore whether the 5′/3′ exon coverage imbalance can be informative in cancer diagnostics.

In this study, the applicability of RNA-seq data 5′/3′ exon coverage imbalance analysis for the detection of *RET* fusion oncogenes was evaluated. A survey of the extant literature reveals that the majority of studies on *RET* 5′/3′ expression imbalance have focused on lung cancer [24,40,41,45,46,47] or on isolated cases of rare tumors [42]. To the best of our knowledge, however, no studies have yet investigated thyroid cancer. Conversely, a pan-cancer analysis of 1327 experimental tumor RNA-seq profiles was conducted, encompassing 221 thyroid cancer cases.

We ascertained that a 5′/3′ RNA-seq *RET* coverage imbalance can effectively predict clinically actionable *RET* fusions with 100% sensitivity and specificity, even in the absence of sequenced chimeric RNA-seq reads. In our sampling, we also identified one extremely rare (*RUFY3::RET*) and two novel (*FN1::RET*, *PPP1R21::RET*) fusions of the *RET* gene. The results of the study demonstrate that exon coverage imbalance analysis is a robust complement to computational RNA-seq pipelines for detecting clinically relevant *RET* fusions.

## 2. Results

### 2.1. RET Coverage Asymmetry Screening

In our previous research [34], we demonstrated that RNA-seq coverage imbalance between the 5′ and 3′ ends of the *ALK* gene could be used to accurately detect its oncogenic rearrangements, thereby extending the utility of whole-transcriptome RNA-seq. In the present study, we sought to ascertain whether the aforementioned strategy could be applied to the detection of oncogenic rearrangements of the *RET* gene.

Preliminary analysis indicates that, according to the COSMIC database [48] (https://cancer.sanger.ac.uk/cosmic; accessed on 19 March 2025) and the FusionGDB 2.0 database [49] (https://compbio.uth.edu/FusionGDB2/; accessed on 19 March 2025), the breakpoint in *RET* 3′ fusions is located in intron 11 in approximately 99% of cases. A similar trend is reported in other published studies [16,21], with approximately 90% of breakpoints located in this region. Consequently, in the presence of an oncogenic *RET* fusion, coverage of exon 12 and downstream exons are expected to be covered to a greater extent than exons located upstream of the breakpoint.

We employed our original approach to assess *RET* coverage asymmetry in our experimental RNA-seq dataset, which included 1248 whole-transcriptome sequencing (WTS) profiles obtained for samples representing different tumor types (Table 1). In each sample, the normalized expression values were calculated for all individual *RET* exons. The normalized expression values were defined as the read coverage normalized to exon length and the total number of reads in the sample. The assessment of *RET* coverage asymmetry was conducted by employing the *p*-value from a one-sided Mann–Whitney U test, which was used to make a comparison between the coverage of exons 12–16 and that of exons 2–6. The choice of exons was based on the following considerations: (i) to ensure sufficient statistical power of differential coverage to reach the 5% significance threshold, and (ii) to confirm the functional integrity of a putative *RET* fusion by confirming the preservation of the tyrosine kinase (TK) domain (Figure 1). Accordingly, the first five exons coding TK domain were used for the TK region coverage calculation, and the same number of upstream exons was selected on the 5′ moiety of the gene, excluding exon 1, which was expected to be underrepresented due to the cDNA priming protocol.

**Figure 2:**
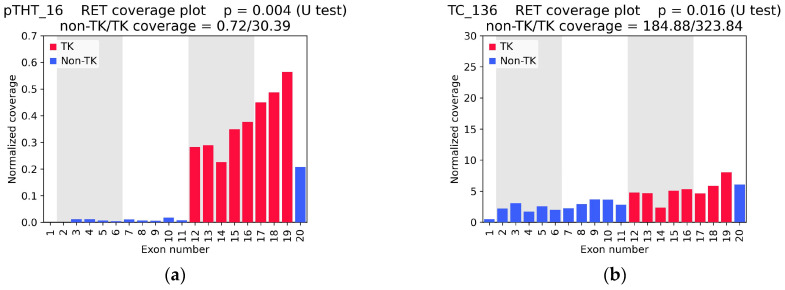

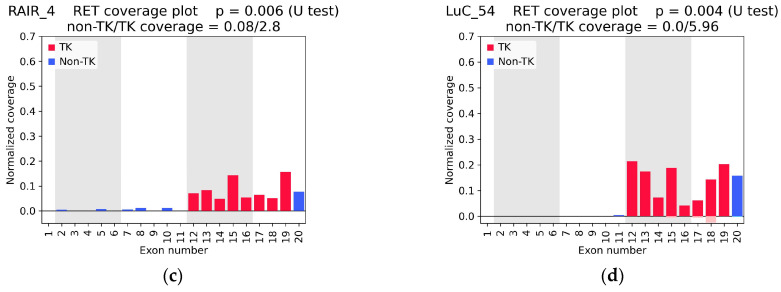
Examples of *RET* coverage plots based on RNA-seq data, normalized to exon length and the total number of reads in each sample. (**a**,**c**,**d**) Samples pTHT_16, RAIR_4, and LuC_54 showed distinct coverage asymmetry, with overexpression of exons 12–19 encoding the tyrosine kinase domain; (**b**) Sample TC_136 showed a statistically significant difference in coverage between the 5′ and 3′ ends of the gene, while maintaining high coverage across all exons. TK—tyrosine kinase domain-related exons; non-TK—exons not related to the tyrosine kinase domain; non-TK/TK coverage—ratio of the mean coverage of five non-TK exons (exons 2–6) to that of five TK exons (exons 12–16).

A total of 197 out of 1248 samples showed experimentally cohort demonstrated asymmetric coverage, as indicated by a U-test *p*-value of less than 0.05. This finding significantly exceeded the anticipated pan-tumor frequency of oncogenic *RET* fusions. This finding likely reflects a high rate of false-positive predictions when relying solely on the *p*-value threshold. Concurrently, it was observed that samples in which junction reads were detected by WTS (Figure 2a, Supplementary Table S1) exhibited a more pronounced difference in 5′/3′ coverage compared with samples devoid of fusion-supporting reads (Figure 2b, Supplementary Table S1). This finding suggests the possibility of RNA-seq coverage asymmetry in the context of wild-type *RET* expression. This observation indicated the necessity of incorporating an additional parameter to account for the extent of variations in coverage between non-TK and TK exons.

In order to further identify coverage characteristics that could help distinguish true positives from false positives, and to test our hypothesis that samples with a statistically significant but modest difference in 5′/3′ coverage reflect expression of wild-type *RET* rather than *RET* fusions, we assessed *RET* status in 78 selected cases using targeted sequencing.

### 2.2. RET Testing with Targeted NGS Panels and Sanger Sequencing

For the purpose of experimental validation, a cohort of 78 tumor samples was analyzed (Table 2). The samples exhibited diverse *RET* coverage profiles (Supplementary Figure S1), spanning high and low expression levels, *p*-values for coverage asymmetry both above and below 0.05, and variable degrees of 5′/3′ coverage imbalance (Supplementary Table S1).

To ascertain *RET* status, a targeted NGS approach was employed, leveraging two specialized panels designed for the identification of fusion transcripts: the TruSight RNA Fusion Panel (Illumina, San Diego, CA USA) and the OncoFu Elite RNA Panel v1.0 (Nanodigmbio, Nanjing, China). Both panels utilize hybridization-based enrichment process. For the OncoFu panel, the same cDNA libraries generated for RNA-seq were used as input. For the TruSight panel, new libraries were prepared directly from tumor tissue RNA samples according to the manufacturer’s protocol. Subsequently, the STAR-Fusion package was employed to identify fusion transcripts in the resulting sequencing reads [50]. In cases where multiple *RET* structural variants were detected, the canonical variant and/or the one with the strongest sequencing read support was selected for further analysis. For samples in which reads supporting a *RET* fusion were directly identified in the WTS RNA-seq data, the putative fusion breakpoint was confirmed by Sanger sequencing.

In 23 tumor samples, a statistically significant *RET* coverage imbalance in WTS RNA-seq reads was identified (*p* < 0.05), as shown in Table 3 and Supplementary Table S1. In the remaining 55/78 samples, neither *RET* coverage asymmetry nor fusion RNA reads were detected by targeted panel NGS.

In the RNA-seq data, junction reads were identified in 11 samples, including 7 cases of pediatric papillary thyroid carcinoma. The confirmation of all detected fusions was achieved through targeted panel sequencing and/or Sanger sequencing (Table 3). In these samples, the *p*-values for 5′/3′ imbalance were found to be less than 0.01, with the 5′/3′ *RET* exon coverage ratio measuring below 0.28.

In one additional specimen of papillary thyroid cancer (RAIR_4), a solitary supporting read was identified, corresponding to the standard *NCOA4(7)::RET(12)* fusion. However, this particular sample exhibited a pronounced *RET* coverage imbalance (Figure 2c). Sanger sequencing has validated the *RET* fusion oncogene in this particular case (Supplementary Figure S2).

Additionally, in one lung adenocarcinoma specimen (LuC_54) exhibiting no WTS RNA-seq fusion reads but with significant *RET* coverage asymmetry, panel sequencing has identified the *KIF5B(15)::RET(12)* fusion (Figure 2d).

The 10 samples that exhibited statistically significant coverage asymmetry but where no fusions were detected by either WTS or targeted sequencing differed from the cases with true confirmed fusions by virtue of their relatively high 5′/3′ *RET* exon coverage ratio (Supplementary Figure S1, Table 3, bottom).

Thus, exon coverage of wild-type *RET* was also somewhat uneven, although to a considerably lesser extent than the 5′/3′ coverage imbalance observed in *RET* fusion-positive samples. In general, variability in exon coverage in whole-transcriptome sequencing can arise from two categories of factors. The first category is biological, and includes factors such as differences between transcript isoforms. The second category is technical, and includes well-known biases in fragment amplification during library preparation or the exclusion of non-uniquely mapped reads. Further studies are necessary to elucidate the underlying causes of uneven wt *RET* coverage. For example, long-read sequencing approaches, which are feasible only with fresh samples containing intact RNA, could be used to achieve this objective.

In light of the findings, an effort was made to delineate a threshold *p*-value and a 5′/3′ *RET* exon coverage ratio that would facilitate the differentiation between true-positive and false-positive samples.

### 2.3. RNA-Seq Coverage Threshold Values for Detection of RET Fusions

**Figure 3:**
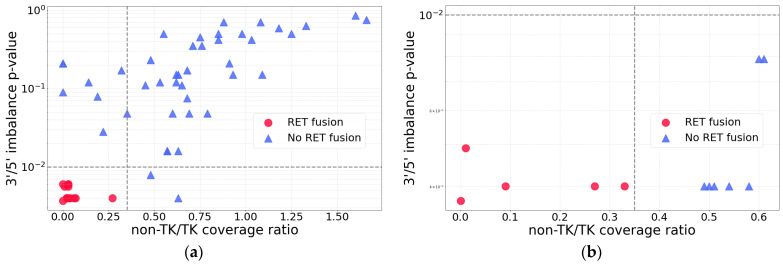
Relationship between *RET* fusion status and RNA-seq coverage asymmetry characteristics in the experimental cohort. Red dots represent cases with *RET* fusions confirmed by targeted NGS panel(s) and/or Sanger sequencing, and blue dots represent cases where no *RET* fusions were detected. Non-TK/TK coverage (5′/3′ *RET* exon coverage ratio)—ratio of the mean coverage of five non-TK exons (exons 2–6) to that of five TK exons (exons 12–16). The selected threshold values are indicated with dashed lines (*p* = 0.01 and non-TK/TK coverage ratio = 0.35). (**a**) Data for the main validation cohort. (**b**) Data for the additional validation cohort.

A systematic comparison of all the cases under analysis with known *RET* status demonstrated lower *p*-values and 5′/3′ *RET* exon coverage ratios for the true *RET* fusion cases (Figure 3a). This approach enabled the establishment of threshold values for *p* = 0.01 and a 5′/3′ *RET* exon coverage ratio of 0.35. Cases exhibiting both parameters below these thresholds were designated as true *RET* fusion cases.

In light of our prior experience with 5′/3′ imbalance analysis of *ALK*, it has been established that inadequate RNA-seq coverage can result in both false-positive and false-negative outcomes [34]. For *ALK*, a minimum coverage depth of 0.7 had previously been established as the threshold for reliable detection based on coverage imbalance [34]. To determine an appropriate threshold for *RET*, RNA-seq data for three fusion-positive samples (pTHT_16, pTHT_21, pTHT_25) were subsampled. From the original FASTQ files, random subsets comprising 2–80% of reads were generated in triplicate for each sample. For each subsample, the *RET* exon 12–16 coverage and U-test *p*-values for asymmetry were calculated. As shown in Supplementary Figure S3, simulated 3′ coverage depths below 1.5 yielded U-test *p*-values that exceeded the selected threshold of 0.01 in true-positive samples, thereby losing statistical significance. These results support the use of 1.5 as the minimum coverage depth necessary for accurate detection of *RET* fusions.

In order to validate the aforementioned findings, the previously established diagnostic thresholds were applied to an additional cohort of samples. To this end, we additionally performed WTS RNA-seq for 79 new thyroid cancer samples (*RET* coverage plots given in Supplementary Figure S4).

According to the predetermined criteria (*p* < 0.01; 5′/3′ *RET* exon coverage ratio < 0.35, 3′ coverage depth > 1.5), *RET* fusions were predicted in four samples (THT_51, THT_53, THT_58, and THT_70; see Supplementary Table S2). In order to assess the accuracy of these predictions, a total of 11 thyroid cancer samples with *p* < 0.01—including the four cases with predicted *RET* fusions—were subjected to further experimental validation using the targeted NGS panel OncoFu and Sanger sequencing. Furthermore, the analysis encompassed one sample (CC_29) from the primary cohort that satisfied the aforementioned criteria but lacked splice junction reads and had not been previously analyzed by targeted sequencing (Supplementary Table S2).

**Figure 4:**
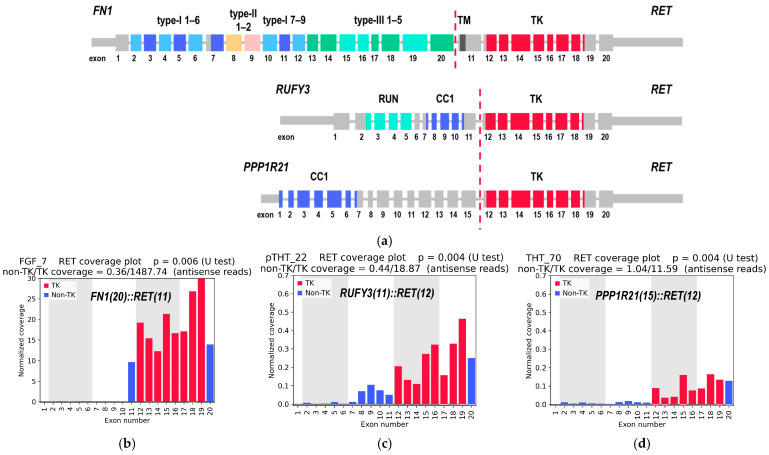
(**a**). Schematic representation of rare *RET* fusions identified in this study. Domains abbreviations for *FN1*: fibronectin type-I (1–9), fibronectin type-II (1–2), and fibronectin type-III (1–5) domains. Domains abbreviations for *RUFY3*: RUN—RUN domain; CC1—coiled-coil domain 1. Domain abbreviation for *PPP1R21*: CC1—coiled-coil domain 1. Domains abbreviation for *RET*: TM—transmembrane domain; TK—tyrosine kinase domain. (**b**–**d**) *RET* coverage plots based on RNA-seq data, normalized to exon length and the total number of reads in each sample: (**b**) sample FGF_7 with *FN1(20)::RET(11)* fusion; (**c**) sample pTHT_22 with *RUFY3(11)::RET(12)* fusion; (**d**) sample THT_70 with *PPP1R21(15)::RET(12)* fusion. TK—tyrosine kinase domain-related exons; non-TK—exons not related to the tyrosine kinase domain; non-TK/TK coverage—ratio of the mean coverage of five non-TK exons (exons 2–6) to that of five TK exons (exons 12–16).

Targeted panel and Sanger sequencing confirmed the presence of *RET* fusions in all predicted samples, while no *RET* fusions were detected in the remaining tested samples (Figure 4b, Supplementary Table S2). Therefore, the selected thresholds are effective in avoiding false-positive predictions arising from overexpression of the wild-type *RET* gene.

It is noteworthy that all medullary thyroid cancer samples (i.e., THT_1–50) expressed wild-type *RET* at a high level (see Supplementary Table S2). *RET* amplification is considered as promising target for anti-RET therapy, along with *RET* rearrangements and activating point mutations [51]. For all pTHT and THT samples, WES profiles were available in addition to WTS data. Therefore, the potential of wild-type *RET* overexpression being explained by gene amplification was investigated. However, CNV analysis did not reveal *RET* amplification in any of the thyroid cancer samples. Consequently, the underlying causes of wild-type *RET* overexpression in medullary thyroid cancer samples, such as aberrations in *RET* regulatory factors, necessitate further investigation.

### 2.4. Rare and Previously Unreported RET Fusions

In the present study, three rare *RET* fusions with preserved open reading frames were identified. Two of these fusions (*FN1(20)::RET(11)* and *PPP1R21(15)::RET(12)*) have not been previously reported in the literature or deposited in public databases, while one (*RUFY3(11)::RET(12)*) has been reported only once, in a different cancer type (Figure 4a). Coverage plots for *RET*, derived from RNA-seq reads for the respective samples, are presented in Figure 4b–d.

These fusions are considered clinically relevant because they maintain an intact open reading frame and conserve the full *RET* tyrosine kinase domain (denoted as TK in Figure 4a), which is essential for kinase activation and therapeutic targeting.

All three 5′ partner genes are located on chromosomes distinct from *RET*: *FN1* (fibronectin 1) on chromosome 2q35, *RUFY3* (RUN and FYVE domain-containing 3) on chromosome 4q13.3, and *PPP1R21* (protein phosphatase 1 regulatory subunit 21) on chromosome 2p16.3.

The *FN1* gene is responsible for encoding fibronectin, a protein that is found in the extracellular matrix and is involved in a variety of essential cellular processes. These processes include wound healing and embryonic morphogenesis. Additionally, fibronectin plays a role in regulating cell behaviors such as adhesion and migration [52]. Fibronectin has also been demonstrated to exhibit self-association properties.

*RUFY3* is a gene belonging to a five-member *RUFY* gene family that is conserved across species. It is expressed in a variety of human tissues and has been implicated in endosomal trafficking, autophagy, and cell migration [53,54].

The *PPP1R21* gene is responsible for encoding a cofactor of protein phosphatase 1 (*PP1*) [55], a key serine/threonine phosphatase that is imperative for several processes within cells, including cell division, glycogen metabolism, protein synthesis, and muscle contractility. *PPP1R21* has been identified as a participant in endosomal maturation and exhibits broad expression in multiple tissues, including the thyroid (https://www.proteinatlas.org/ENSG00000162869-PPP1R21/tissue, accessed on 11 September 2025).

In the FGF23-producing adenoma sample (FGF_7), a fusion transcript involving exon 20 of *FN1* and exon 11 of *RET* genes was detected (Figure 4a). In this case, RET activation may ensue as a consequence of its fusion with a gene that is driven by a constitutively active promoter. This process ultimately leads to the overproduction of the *RET* tyrosine kinase domain. Furthermore, since the fusion also retains *FN1* domains critical for intermolecular interactions (type I repeats 1–5 and type III repeat 1) [52], activation is likely mediated through oligomerization of the fusion protein. It is noteworthy that this fusion maintained the integrity of the *RET* transmembrane domain, a component that frequently becomes lost during rearrangements. This observation suggests that the *FN1(20)::RET(11)* fusion product may have the potential to localize within the membrane. Although previous studies have reported various fusions involving the *FN1* gene and different tyrosine kinase genes [56,57,58], only a single case of a *FN1::RET* fusion has been described. This case involved a different tumor type, namely pseudosarcomatous myofibroblastic proliferation of the urinary bladder, and different partner exons, namely *FN1(28)::RET(6)* [59].

In turn, the activation of RET in the rearrangements involving *RUFY3* and *PPP1R21* is most likely mediated by ligand-independent dimerization of these fusion proteins, since both 5′ partner moieties retain coiled-coil domains (Figure 4a). To the best of our knowledge, the *RUFY3(11)::RET(12)* fusion has hitherto been reported only once, in a non-small cell lung cancer specimen [60]. In the present study, the presence of the aforementioned element was detected in a sample of pediatric papillary thyroid cancer (pTHT_22). A *RET* fusion with *RUFY3* was also mentioned in [61], described as identified through the analysis of three public datasets (TCGA-THCA, MSK-MetTropism, and MSK-IMPACT), although no further details were provided.

The *PPP1R21::RET* rearrangement has been previously reported in the context of thyroid cancer, albeit with a distinct breakpoint, namely *PPP1R21(9)::RET(12)* [60]. In the present study, the breakpoint in *PPP1R21* was identified as occurring subsequent to exon 15.

## 3. Discussion

Despite the growing clinical importance of detecting fusions involving various tyrosine kinases, driven by the increasing availability of highly effective targeted therapies, their identification in routine practice remains challenging. This phenomenon is primarily attributable to the exceedingly low frequency of fusions for each individual gene, in conjunction with the substantial diversity of potential partner genes and possible breakpoint locations. Conventional methods, including IHC and FISH, are inadequate for this purpose due to their reliance on the specific fusion partner involved, which often compromises the accuracy and precision of the results. Furthermore, these approaches are deficient in multiplexing capability and are susceptible to errors resulting from the substantial subjectivity inherent in data interpretation.

In this context, high-throughput methods capable of detecting fusions in a partner-gene-agnostic manner across large panels or even genome-wide have become indispensable. These include targeted sequencing panels and whole-transcriptome sequencing (WTS, RNA-seq). The former is more widely adopted in clinical practice due to its lower cost, higher coverage of target loci, and simpler bioinformatic analysis. Concurrently, while WTS is associated with a higher financial investment and a more intricate analytical framework, it offers a remarkably comprehensive array of information concerning the molecular characteristics of tumors [62].

At the same time, the sensitivity of WTS for fusion detection is relatively limited, as only a few reads typically span the fusion junction. We previously demonstrated that the sensitivity of *ALK* fusion detection in RNA-seq data increases to 100% when, in addition to chimeric reads, exon expression profiles are analyzed to identify 5′/3′ coverage imbalance [34]. In the present study, the efficacy of this approach in detecting *RET* fusions was assessed.

An investigation was conducted to identify *RET* coverage disparities in a substantial collection of experimental RNA-seq data (*n* = 1327 profiles, comprising 154 non-small cell lung cancer and 221 thyroid cancer samples). Subsequent to this screening process, the validity of *RET* status was confirmed in 78 selected cases exhibiting diverse *RET* coverage patterns. To confirm the *RET* status, targeted panel sequencing and Sanger sequencing were employed. The selection of threshold values for the *p*-value and the 5′/3′ *RET* exon coverage ratio was made based on the obtained data. The purpose of this selection was to allow for the distinction between true-positive and false-positive predictions. The selected threshold parameters for *RET* coverage imbalance in RNA-seq were subsequently validated in an independent cohort of 79 thyroid cancer samples.

While the majority of the *RET* fusions identified in the experimental cohort (11 out of 14) exhibited junction reads in the RNA-seq data, *RET* coverage asymmetry analysis facilitated the detection and validation of *RET* fusions in three additional samples (RAIR_4, LuC_54, and CC_29) among a total of more than 1300 samples examined. Therefore, the analysis of *RET* coverage asymmetry in RNA-seq data has the potential to facilitate the detection of extremely rare fusion events, even in cases of low coverage depth. The sensitivity and specificity of the 5′/3′ *RET* exon coverage imbalance analysis of RNA-seq data were both 100%, as determined by the selected 5′/3′ *RET* exon coverage ratio and *p*-value thresholds.

It is important to note that the proposed approach can be applied to different tumor types. As *RET* not typically expressed in lung tissue, *RET* coverage imbalance has been identified as a reliable marker of fusion presence in lung cancer, as confirmed by previously published studies [24,40,41,45,46,47]. However, in the context of thyroid cancer, a notable exception emerges, as numerous samples manifest an elevated expression of wild-type *RET*. It was determined that such samples also manifest a statistically significant 5′/3′ coverage imbalance, with pronounced differences between 5′ and 3′ *RET* exon coverage. This can result in a high rate of false-positive predictions when relying solely on this method. However, the utilization of the established threshold values for the assessment of coverage enables the differentiation of true fusion-positive cases from those exhibiting high wild-type *RET* expression.

The described approach has been observed to be deficient in its inability to identify the fuse partner or the precise breakpoint within the target gene, although in most cases, the latter can be inferred from the exon coverage profile. It should be noted that IHC and FISH methods are similarly incapable of determining the partner gene and exact breakpoints and this information is not currently required for therapeutic decision-making.

According to the ESMO recommendations, NGS is the preferred method over IHC and FISH, and targeted RNA-based assays are the methods of choice for *RET* fusion screening [26]. While whole-transcriptome RNA sequencing is not a first-line diagnostic tool due to its higher cost and analytical complexity, it is increasingly applied in research and in diagnostically challenging clinical cases. In such settings, coverage imbalance analysis can serve as an additional interpretive layer that increases the diagnostic yield of existing WTS data. When exon coverage asymmetry is detected, confirmatory testing using targeted NGS panels or anchored PCR-based NGS can be employed to identify the fusion partner and confirm precise breakpoint. The proposed method increases the practical utility and cost-effectiveness of WTS by enabling the identification of clinically actionable *RET* fusions that might otherwise remain undetected.

Therefore, we propose that the detection of *RET* exon coverage asymmetry can serve as an independent, highly accurate clinical testing procedure. Nonetheless, given the nascent stage of technology adoption, we suggest a workflow that aligns with current practices. Initially, a 5′/3′ coverage imbalance analysis should be conducted on the RNA-seq data. In instances where the imbalance analysis predicts the presence of *RET* fusion, the subsequent step involves validating the results using established methods (Supplementary Figure S5).

In the present study, we observed significant overexpression of wild-type *RET* in all medullary thyroid cancer samples (THT_1–50). Although gene amplification is considered another potential targetable alteration for anti-RET therapy [51], WES analysis did not reveal *RET* amplification in these cases. According to the extant data, *RET* overexpression occurs in 40–60% of breast tumors and it is reported to be more common than *RET* rearrangements or mutations in breast cancer [63]. *RET* overexpression typically correlates with elevated protein levels [63]. However, extant research on this aspect remains largely limited to preclinical cell-based studies [64,65]. Nonetheless, *RET* overexpression, irrespective of amplification status, may signify an ancillary opportunity for the implementation of selective RET inhibitors. These should be evaluated in conjunction with *RET* rearrangements and activating mutations, for which RNA-seq is similarly informative. A further imperative is to undertake a thorough investigation into the mechanisms that underlie *RET* overexpression in the absence of gene amplification.

Among the 18 *RET* fusion-positive samples identified in this study, we detected one rare fusion event (*RUFY3(11)::RET(12)*) as well as two new *RET* fusions that had not been previously described in the literature (*FN1(20)::RET(11)* and *PPP1R21(15)::RET(12)*).

In summary, the present study demonstrated the efficacy of exon coverage imbalance analysis as a substitute for other *RET* fusion detection methods. The present study offers a valuable complement to RNA sequencing data analysis pipelines, thereby enabling the effective detection of clinically relevant oncogenic *RET* alterations.

## 4. Materials and Methods

### 4.1. Biosamples and RNA Sequencing

In this study, experimental whole-transcriptome data obtained for 1327 tumor samples representing 28 cancer types and 2 benign tumor types were used. The tumor tissue samples were either obtained during the course of the Oncobox clinical trial (Clinicaltrials.gov ID NCT03724097, NCT03521245) (*n* = 764) or obtained from individual patients who submitted their biosamples for Oncobox molecular testing. For each biospecimen, written informed consent for participation in this study was obtained from the patient or the patient’s legal representative. The consent process and study design were guided and approved by the local ethics committees of the Vitamed Clinic (Moscow, Russia). In addition, 113 thyroid tissue specimens were obtained from the National Medical Research Center for Endocrinology (Moscow, Russia). The study was approved by the local ethics committees of the Vitamed Clinic, Moscow, Russia (protocol #1, approval date: 16 October 2017), and the National Medical Research Center for Endocrinology, Moscow, Russia (protocol #1, approval date: 15 January 2025).

The samples were formalin-fixed paraffin-embedded (FFPE) tissue blocks that were macrodissected for tumor-rich regions identified on hematoxylin and eosin (H&E)-stained slides. RNA extraction, library preparation, sequencing, and RNA-seq data processing were performed as described in [25,66], and the resulting BAM files were then analyzed.

Most NGS libraries were sequenced on either the HiSeq 3000 or the NextSeq 550 platforms (both from Illumina, San Diego, CA, USA) in single-end mode, with read lengths of 50 bp and 75 bp, respectively. More recent samples were sequenced on the FASTASeq 300 (GeneMind Biosciences, Shenzhen, China) with paired read lengths of 75 bp. In total, 1327 RNA-seq tumor profiles had more than the threshold of 2.5 million uniquely mapped reads (Table 1) [25,66]. The gender and age characteristics of all cohorts are shown in Table 1.

### 4.2. Exon Coverage Calculation

RNA-seq reads were aligned to the human genome (hg38, GCF_000001405.39) using STAR v2.7.4a, and duplicate reads were removed using the Picard MarkDuplicates tool with the default parameters. Only reads with a mapping quality (MQ) > 100 and an alignment length ≥ 10 bp were considered. Exon coordinates were obtained from Ensembl (for ENST00000355710.8), and coverage was normalized to both exon length and to the total number of mapped reads. Strand specificity was taken into account. Coverage imbalance for *RET* was assessed by comparing the 5′ non-kinase region exons (2–6) with the tyrosine kinase domain exons (12–16) using a one-sided Mann–Whitney U test with the alternative hypothesis that the coverage of non-TK-related exons is lower than that of TK-related exons. Statistical significance was determined based on the *p*-value. The Mann–Whitney U test was used due to the non-normality of the exon coverage distribution, as assessed by the Shapiro–Wilk test for each sample (with the null hypothesis that the distribution is normal).

The coverage depth of *RET* exons 12–16 was calculated by dividing the total number of aligned bases by the combined exon length. Samples with undetectable or insufficient *RET* transcript coverage were excluded from the analysis. The threshold for low coverage is described in the Results section.

The code used for the analysis and the accompanying data are available at https://github.com/akhristichenko/RET_coverage_asymmetry. (accessed on 5 October 2025).

### 4.3. Experimental Validation of RET Fusion Transcripts by Targeted NGS Panels

Two targeted NGS panels were used for *ALK* rearrangement verification: the TruSight RNA Fusion Panel (Illumina, San Diego, CA, USA) and the OncoFu Elite (for RNA) Panel v1.0 (Nanodigmbio, Nanjing, China). Both panels utilize hybridization-based enrichment and cover all coding regions of transcripts, as well as selected untranslated regions (UTRs) of genes that are most frequently or clinically implicated in fusions in solid tumors. These panels enable the detection of both known and previously unreported fusions.

NGS libraries were prepared according to the manufacturer’s protocol. For the TruSight panel, libraries were prepared using previously extracted RNA samples. For the OncoFu panel, hybridization enrichment was performed on cDNA libraries that had been prepared previously and were used for whole transcriptome sequencing.

Sequencing was performed on the FASTASeq 300 platform (GeneMind Biosciences, Shenzhen, China) with 2 × 75 bp paired-end reads. The expected yield was approximately 4.5 million reads per sample for the TruSight panel and 2.5 million reads per sample for the OncoFu panel.

### 4.4. Experimental Validation of RET Fusion Transcripts by RT-PCR and Sanger Sequencing

To generate amplicons for Sanger sequencing, single-stranded cDNA was synthesized from 100 ng of total RNA using the MMLV RT kit (Evrogen, Moscow, Russia), according to the manufacturer’s instructions. The reverse transcriptase was pre-diluted tenfold with enzyme dilution buffer. Reverse transcription was performed using a mix of gene-specific reverse primers for *RET* exons 11 and 12 (5′-GACAGCAGCACCGAGAC-3′ and 5′-CAAGAACCAAGTTCTTCCGAG-3′), at a final concentration of 500 nM.

The following reagents were used for preparative PCR (all from Evrogen, Moscow, Russia): 10× Turbo Buffer, a dNTP mix (10 mM each), HS-Taq DNA polymerase, and PCR-grade water. PCR was performed in 50 µL volumes using a T100 Thermal Cycler (Bio-Rad, Hercules, CA, USA). The thermal cycling protocol was as follows: (1) 2 min at 95 °C; (2) 40 cycles of 15 s at 95 °C, 20 s at 60 °C, 10 s at 70 °C; (3) 1 min at 70 °C.

The primer design was performed as follows: (1) the region surrounding the fusion breakpoint (150 bp upstream and downstream) was examined for paralogous sequences using BLAT (https://genome-euro.ucsc.edu/cgi-bin/hgBlat?hgsid=324308355_bHclKcKtEyJWfpBYXjHTsAh5QF7h&command=start; accessed on 8 September 2025); (2) primers were designed so that the 3′ end would align to regions without detectable homology; (3) the forward primer was placed on the last exon of the 5′ fusion partner gene immediately upstream of the breakpoint, and the reverse primer was placed on the first exon of RET, immediately downstream of the breakpoint; (4) due to the degraded nature of RNA in FFPE samples, amplicon lengths were kept between 100 and 150 bp; (5) primers were designed with a melting temperature of 61 ± 1 °C and were checked to avoid hairpin formation, homodimer formation, and heterodimer formation using the OligoAnalyzer tool (https://www.idtdna.com/pages/tools/oligoanalyzer; accessed on 8 September 2025). Primer pairs are listed in Table 4.

To verify PCR specificity, negative controls included a non-template control and cDNA from a *RET* fusion-negative tumor sample, neither of which showed amplification.

Amplicons were purified using the standard NGS Clean and Select Beads protocol (Meridian Bioscience, Cincinnati, OH, USA) with a twofold excess of the volume of the magnetic bead suspension. Sanger sequencing was performed using a Genetic Analyzer 3500xL (Applied Biosystems, Foster City, CA, USA).

### 4.5. Bioinformatic Detection of RET Fusion Transcripts

The reads quality was assessed with FastQC (https://github.com/s-andrews/FastQC; accessed on 11 May 2025), and reads were filtered using PRINSEQ-lite (https://github.com/uwb-linux/prinseq; accessed on 11 May 2025) (mean Phred quality ≥ 30).

*RET* fusion transcripts were identified in RNA-seq profiles and data from targeted NGS panels using STAR-Fusion software [50] (version STAR-2.7.2b; https://github.com/STAR-Fusion/STAR-Fusion; accessed on 23 May 2025) with the pre-built CTAT plug-and-play reference GRCh38_gencode_v22_CTAT_lib_Mar012021. Default parameters were used unless otherwise specified.

Fusion candidate files were generated and the relevant RNA sequencing reads associated with *RET* were extracted. The resulting data were verified through manual inspection using UCSC BLAT and the UCSC Genome Browser (https://genome-euro.ucsc.edu, accessed 3 June 2025). Candidate *RET* fusions were evaluated based on the following criteria: (i) whether the sequencing read spans an exon junction between two distinct, previously characterized transcripts; (ii) whether the junction site precisely corresponds to the boundaries of exons of known genes, taking established splice sites into account; and (iii) whether both transcripts align in the same orientation, indicating the presence of a putative fusion RNA. Reads that met these criteria were considered evidence of fusion.

### 4.6. Statistical Analysis

The statistical significance of differences between the groups was estimated using the log-rank test, with the ‘logrank’ function from the Python 3 ‘scipy’ library (https://github.com/scipy/scipy; accessed on 5 October 2025). A *p*-value less than 0.05 was considered to be statistically significant.

## Figures and Tables

**Table 1:** Clinical characteristics of the included tumor cases.

Tumor Type ^1^	Total Cases Sequenced, *n* (%)	Gender, Male/Female	Age of Onset		
			Mean	Median	Range
TC	221 (16.7%)	21.3%/74.7% ^2^	42.7	44.5	6–81
CRC	164 (12.4%)	43.9%/56.1%	59.0	59	32–87
NSCLC	154 (11.6%)	68.2%/31.8%	60.7	62.5	29–81
BC	147 (11.1%)	0%/100%	53.0	54	27–89
CNS	82 (6.2%)	57.3%/36.6% ^2^	39.8	40	3–70
OC	80 (6.0%)	0%/100%	52.3	52	27–80
SC	67 (5.0%)	55.2%/44.8%	57.4	59	29–81
PC	61 (4.6%)	55.7%/44.3%	60.2	62	31–79
MM	59 (4.2%)	55.9%/42.4% ^2^	58.4	59	29–78
AL	50 (3.8%)	54.0%/46.0%	5.8	5	1–15
KC	37 (2.6%)	67.6%/32.4%	56.0	57	40–72
Other	211 (15.9%)	37.9%/60.7% ^2^	51.6	52	14–83

^1^ Tumor type abbreviations: NSCLC—non-small-cell lung cancer; CRC—colorectal cancer; TC—thyroid cancer; BC—breast cancer; CNS—central nervous system cancer; MM—multiple myeloma; AL—acute lymphoblastic/myeloid leukemia; OC—ovarian cancer; SC—stomach cancer; PC—pancreatic cancer; KC—kidney cancer; SCLC—small-cell lung cancer. ^2^ For the remaining several samples, the annotation was lost.

**Table 2:** Tumor samples used for validation of *RET* rearrangement detection.

Tumor Type	Number of Samples	Number of Samples Tested with		
		TruSight NGS	OncoFu NGS	Sanger
Breast cancer	4	4	4	0
Cervical cancer	1	1	1	0
Cholangiocarcinoma	2	0	2	0
FGF23-producing adenoma	1	0	0	1
Fibrosarcoma	1	0	0	1
Glioblastoma, IDH wild type	2	1	2	0
Hemangioendothelioma	2	0	2	0
Leukemia	5	5	5	0
Liposarcoma	1	1	1	0
Lung cancer	24	22	23	0
Melanoma	1	1	1	0
Mesothelioma	1	1	1	0
Mucoepidermoid carcinoma	1	0	1	0
Ovarian cancer	4	3	4	0
Pancreatic cancer	3	3	3	0
Papillary thyroid cancer	19	2	12	8
Parathyroid cancer	1	0	1	0
Prostate cancer	1	0	1	0
Rectal cancer	3	2	3	0
Salivary gland cancer	1	1	1	0
Overall	78	47	68	10

**Table 3:** Results of *RET* fusion validation using the TruSight and OncoFu targeted NGS panels.

Sample ID	RNA-Seq Coverage Characteristics			Found *RET* Fusion (Number of Junction and Spanning Reads) ^2^			Confirmed by Sanger Sequencing
	5′/3′ Asymmetry *p*-Value	TK Coverage Depth	Non-TK/TK ^1^ Coverage Ratio	RNA-Seq	TruSight	OncoFu	
CC_162	0.006	8.11	0.03	*NCOA4(9)::RET(12)* (6 + 11) ^2^	-	*NCOA4(9)::RET(12)* (329 + 423)	-
FGF_7	0.006	1 487.74	0.00	*FN1(20)::RET(11)* (937 + 267)	-	-	yes
FS_1	0.004	8.90	0.06	*CCDC6(1)::RET(12)* (3 + 0)	-	-	yes
LuC_100	0.006	6.85	0.03	*KIF5B(16)::RET(12)* (5 + 0)	*KIF5B(16)::RET(12)* (10 + 0)	*KIF5B(16)::RET(12)* (93 + 59)	-
pTHT_15	0.0056	29.42	0.01	*CCDC6(1)::RET(12)* (18 + 4)	-	-	yes
pTHT_16	0.004	30.39	0.02	*CCDC6(1)::RET(12)* (31 + 5)	-	-	yes
pTHT_21	0.004	12.44	0.07	*NCOA4(7)::RET(12)* (16 + 1)	-	-	yes
pTHT_22	0.004	18.87	0.02	*RUFY3(11)::RET(12)* (13 + 2)	-	-	yes
pThT_24	0.004	9.35	0.27	*NCOA4(7)::RET(12)* (6 + 0)	-	*NCOA4(7)::RET(12)* (52 + 45)	yes
pTHT_25	0.004	15.83	0.04	*CCDC6(1)::RET(12)* (10 + 1)	-	-	yes
pTHT_26	0.004	20.39	0.03	*TRIM27(3)::RET(12)* (15 + 5)	-	-	yes
RAIR_4	0.0056	2.80	0.03	*NCOA4(7)::RET(12)* (1 + 0)	-	-	yes
LuC_54	0.0037	5.96	0.00	no fusion	*KIF5B(15)::RET(12)* (68 + 10)	*KIF5B(15)::RET(12)* (12 + 4)	-
BC_100	0.048	6.94	0.79	no fusion	no fusion	no fusion	-
OC_49	0.048	1.97	0.69	no fusion	no fusion	no fusion	-
OC_80	0.028	1.24	0.22	no fusion	-	no fusion	-
pTHT_4	0.016	7.26	0.63	no fusion	-	no fusion	-
pTHT_5	0.004	3.38	0.63	no fusion	-	no fusion	-
pTHT_6	0.048	3.19	0.60	no fusion	-	no fusion	-
pTHT_19	0.0079	9.82	0.48	no fusion	-	no fusion	-
pTHT_32	0.016	6.77	0.57	no fusion	-	no fusion	-
TC_136	0.016	323.84	0.57	no fusion	-	no fusion	-
SgC_2	0.048	1.04	0.35	no fusion	no fusion	no fusion	-

^1^ TK—*RET* tyrosine kinase-associated exons 12–16; non-TK—*RET* exons 2–6. ^2^ The numbers in parentheses denote the counts of junction and spanning reads as determined by STAR-Fusion. Junction reads directly span the fusion breakpoints, whereas spanning pairwise reads map to opposite sides of a chimeric transcript without overlapping the breakpoint, both evidencing the presence of the predicted fusion.

**Table 4:** List of primers used to amplify the fusion transcript fragment around the junction point.

Target Fusion	Forward Primer, 5′–3′	Reverse Primer, 5′–3′
*CCDC6(1)::RET(12)*	TGGAGACCTACAAACTGAAGTG	CAAGAACCAAGTTCTTCCGAG
*FN1(20)::RET(11)*	CCCAAAGCCACTGGAGTC	GACAGCAGCACCGAGAC
*NCOA4(7)::RET(12)*	ACCTGCCAGTGGTTATCAAG	CAAGAACCAAGTTCTTCCGAG
*PPP1R21(15)::RET(12)*	GTGGATTCATTAGTCCTCTTTCAG	CAAGAACCAAGTTCTTCCGAG
*RUFY3(11)::RET(12)*	GCAGGATGCCCTGGTATC	CAAGAACCAAGTTCTTCCGAG
*TRIM27(3)::RET(12)*	CATCTCCCACCTCAGCAG	CAAGAACCAAGTTCTTCCGAG

## Data Availability

The original contributions presented in this study are included in the article/supplementary material. Further inquiries can be directed to the corresponding author(s).

## Supplementary Materials

The following supporting information can be downloaded at: https://www.mdpi.com/article/10.3390/ijms262311300/s1.
