# Supplementary material for: Accurate RET Fusion Detection in Solid Tumors Using RNA Sequencing Coverage Imbalance Analysis
Source: Int J Mol Sci. 2025 Nov 22;26(23):11300. doi: 10.3390/ijms262311300 (PMC12692729; doi:10.3390/ijms262311300)
Supplement: Supplementary file 1 [file ijms-26-11300-s001.zip › RET_Supplementary Figures_S1-5_20251114.pdf]

Article "Accurate *RET* Fusion Detection in Solid Tumors by RNA Sequencing Coverage Imbalance Analysis"

Supplementary Figures

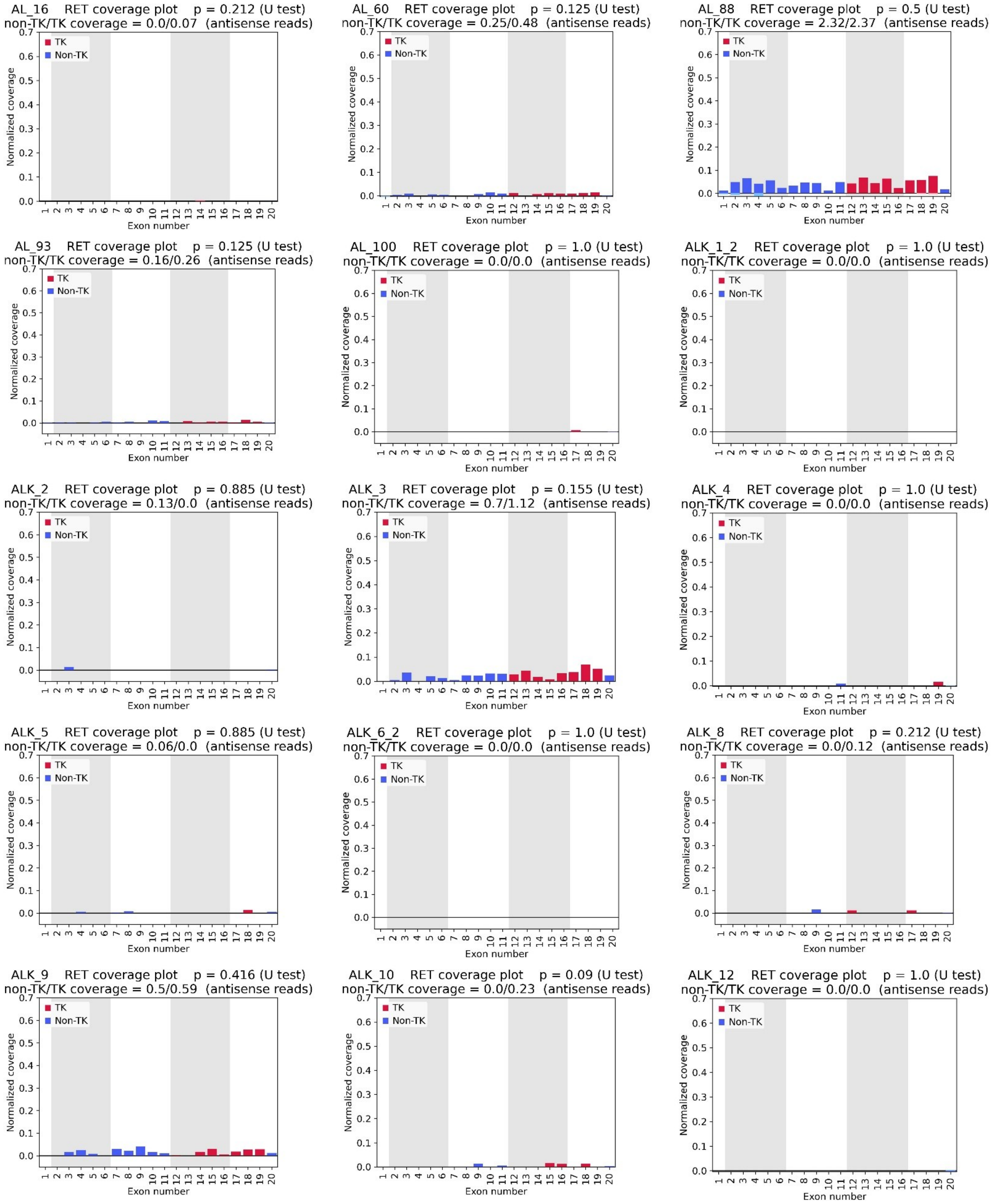

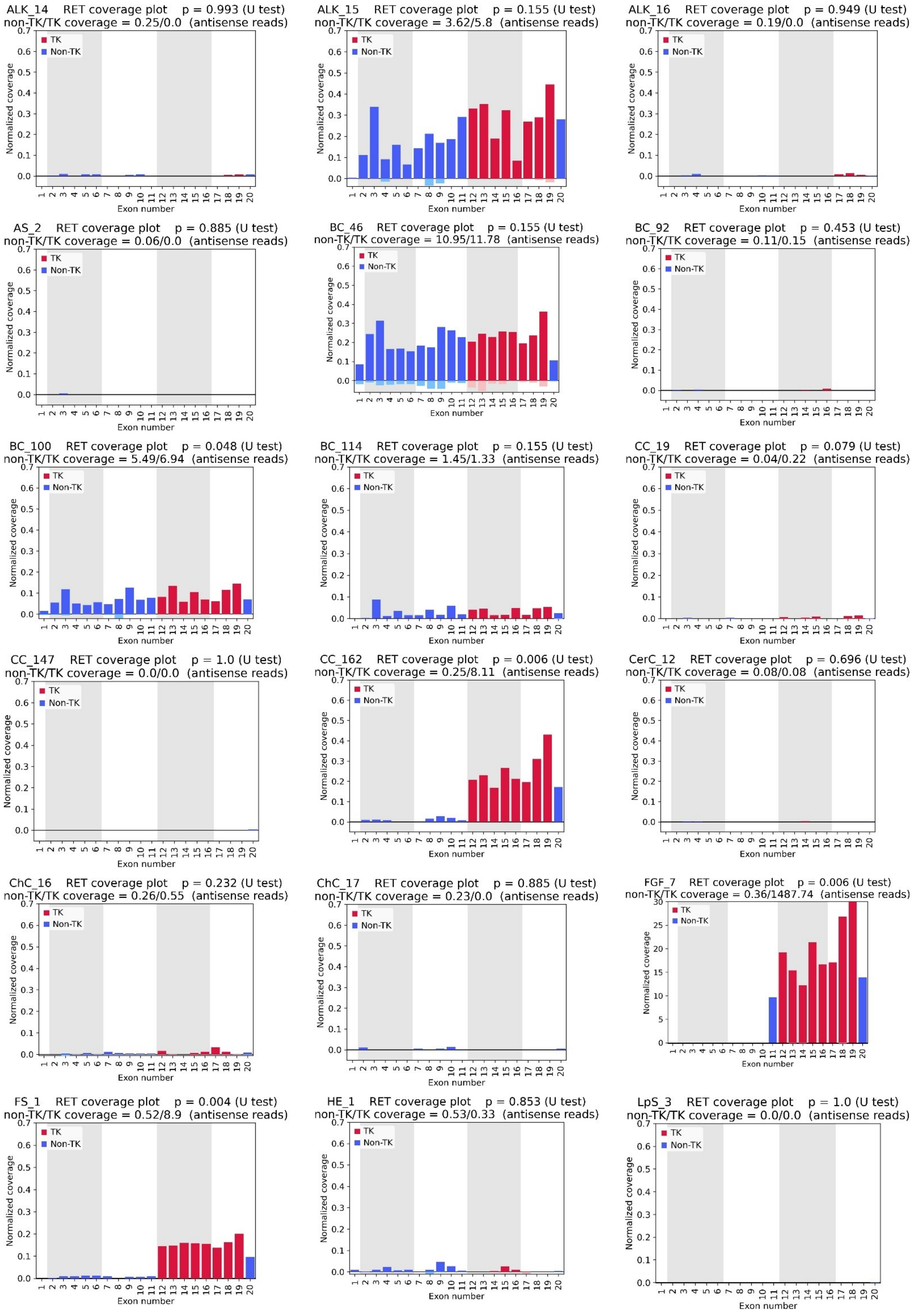
ALK_14 RET coverage plot p = 0.993 (U test)
non-TK/TK coverage = 0.25/0.0 (antisense reads)
ALK_15 RET coverage plot p = 0.155 (U test)
non-TK/TK coverage = 3.62/5.8 (antisense reads)
ALK_16 RET coverage plot p = 0.949 (U test)
non-TK/TK coverage = 0.19/0.0 (antisense reads)
AS_2 RET coverage plot p = 0.885 (U test)
non-TK/TK coverage = 0.06/0.0 (antisense reads)
BC_46 RET coverage plot p = 0.155 (U test)
non-TK/TK coverage = 10.95/11.78 (antisense reads)
BC_92 RET coverage plot p = 0.453 (U test)
non-TK/TK coverage = 0.11/0.15 (antisense reads)
BC_100 RET coverage plot p = 0.048 (U test)
non-TK/TK coverage = 5.49/6.94 (antisense reads)
BC_114 RET coverage plot p = 0.155 (U test)
non-TK/TK coverage = 1.45/1.33 (antisense reads)
CC_19 RET coverage plot p = 0.079 (U test)
non-TK/TK coverage = 0.04/0.22 (antisense reads)
CC_147 RET coverage plot p = 1.0 (U test)
non-TK/TK coverage = 0.0/0.0 (antisense reads)
CC_162 RET coverage plot p = 0.006 (U test)
non-TK/TK coverage = 0.25/8.11 (antisense reads)
CerC_12 RET coverage plot p = 0.696 (U test)
non-TK/TK coverage = 0.08/0.08 (antisense reads)
ChC_16 RET coverage plot p = 0.232 (U test)
non-TK/TK coverage = 0.26/0.55 (antisense reads)
ChC_17 RET coverage plot p = 0.885 (U test)
non-TK/TK coverage = 0.23/0.0 (antisense reads)
FGF_7 RET coverage plot p = 0.006 (U test)
non-TK/TK coverage = 0.36/1487.74 (antisense reads)
FS_1 RET coverage plot p = 0.004 (U test)
non-TK/TK coverage = 0.52/8.9 (antisense reads)
HE_1 RET coverage plot p = 0.853 (U test)
non-TK/TK coverage = 0.53/0.33 (antisense reads)
LpS_3 RET coverage plot p = 1.0 (U test)
non-TK/TK coverage = 0.0/0.0 (antisense reads)
TK
Non-TK
Normalized coverage
Exon number

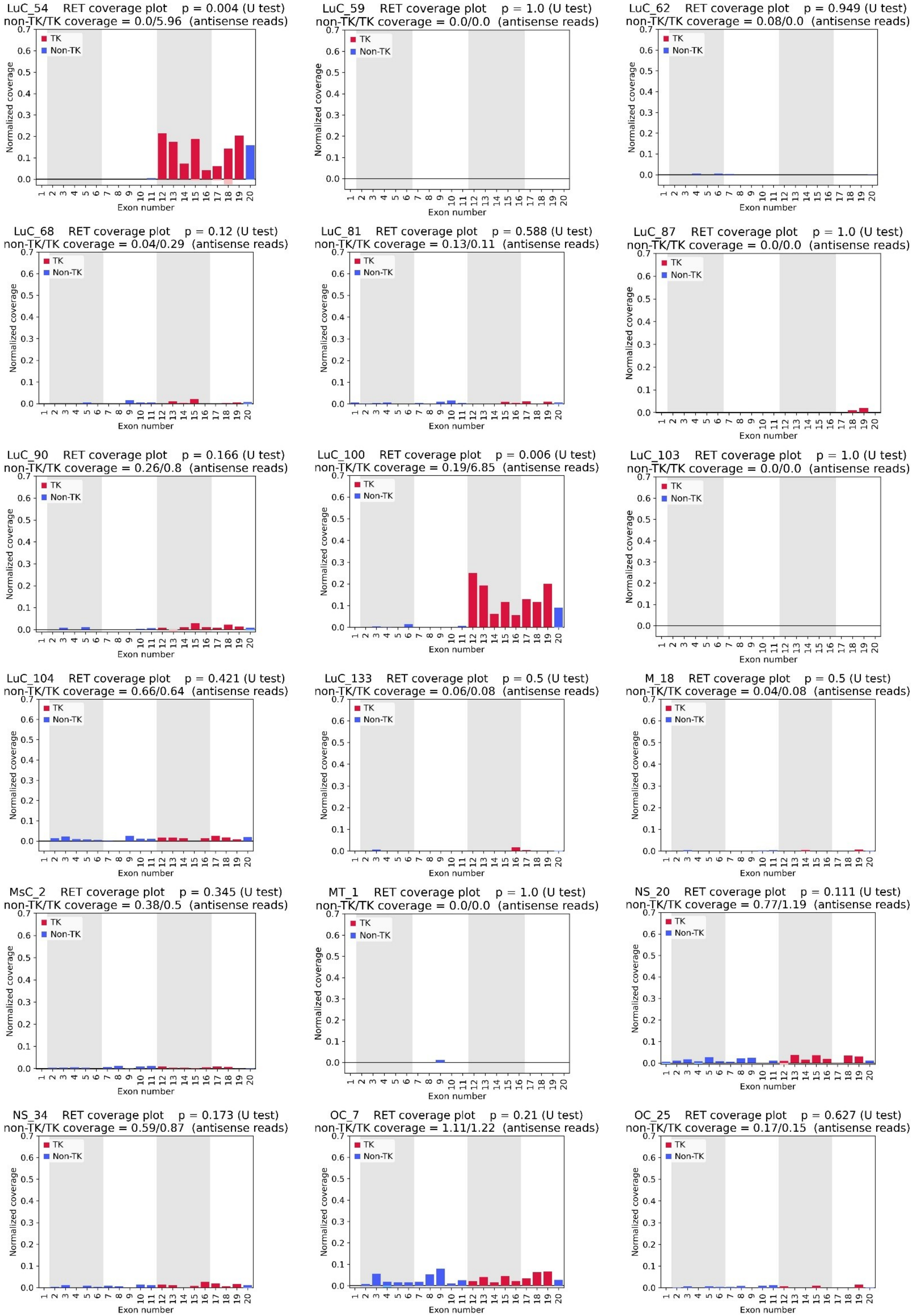
LuC_54 RET coverage plot p = 0.004 (U test)
non-TK/TK coverage = 0.0/5.96 (antisense reads)
LuC_59 RET coverage plot p = 1.0 (U test)
non-TK/TK coverage = 0.0/0.0 (antisense reads)
LuC_62 RET coverage plot p = 0.949 (U test)
non-TK/TK coverage = 0.08/0.0 (antisense reads)
LuC_68 RET coverage plot p = 0.12 (U test)
non-TK/TK coverage = 0.04/0.29 (antisense reads)
LuC_81 RET coverage plot p = 0.588 (U test)
non-TK/TK coverage = 0.13/0.11 (antisense reads)
LuC_87 RET coverage plot p = 1.0 (U test)
non-TK/TK coverage = 0.0/0.0 (antisense reads)
LuC_90 RET coverage plot p = 0.166 (U test)
non-TK/TK coverage = 0.26/0.8 (antisense reads)
LuC_100 RET coverage plot p = 0.006 (U test)
non-TK/TK coverage = 0.19/6.85 (antisense reads)
LuC_103 RET coverage plot p = 1.0 (U test)
non-TK/TK coverage = 0.0/0.0 (antisense reads)
LuC_104 RET coverage plot p = 0.421 (U test)
non-TK/TK coverage = 0.66/0.64 (antisense reads)
LuC_133 RET coverage plot p = 0.5 (U test)
non-TK/TK coverage = 0.06/0.08 (antisense reads)
M_18 RET coverage plot p = 0.5 (U test)
non-TK/TK coverage = 0.04/0.08 (antisense reads)
MsC_2 RET coverage plot p = 0.345 (U test)
non-TK/TK coverage = 0.38/0.5 (antisense reads)
MT_1 RET coverage plot p = 1.0 (U test)
non-TK/TK coverage = 0.0/0.0 (antisense reads)
NS_20 RET coverage plot p = 0.111 (U test)
non-TK/TK coverage = 0.77/1.19 (antisense reads)
NS_34 RET coverage plot p = 0.173 (U test)
non-TK/TK coverage = 0.59/0.87 (antisense reads)
OC_7 RET coverage plot p = 0.21 (U test)
non-TK/TK coverage = 1.11/1.22 (antisense reads)
OC_25 RET coverage plot p = 0.627 (U test)
non-TK/TK coverage = 0.17/0.15 (antisense reads)
TK
Non-TK
Normalized coverage
Exon number

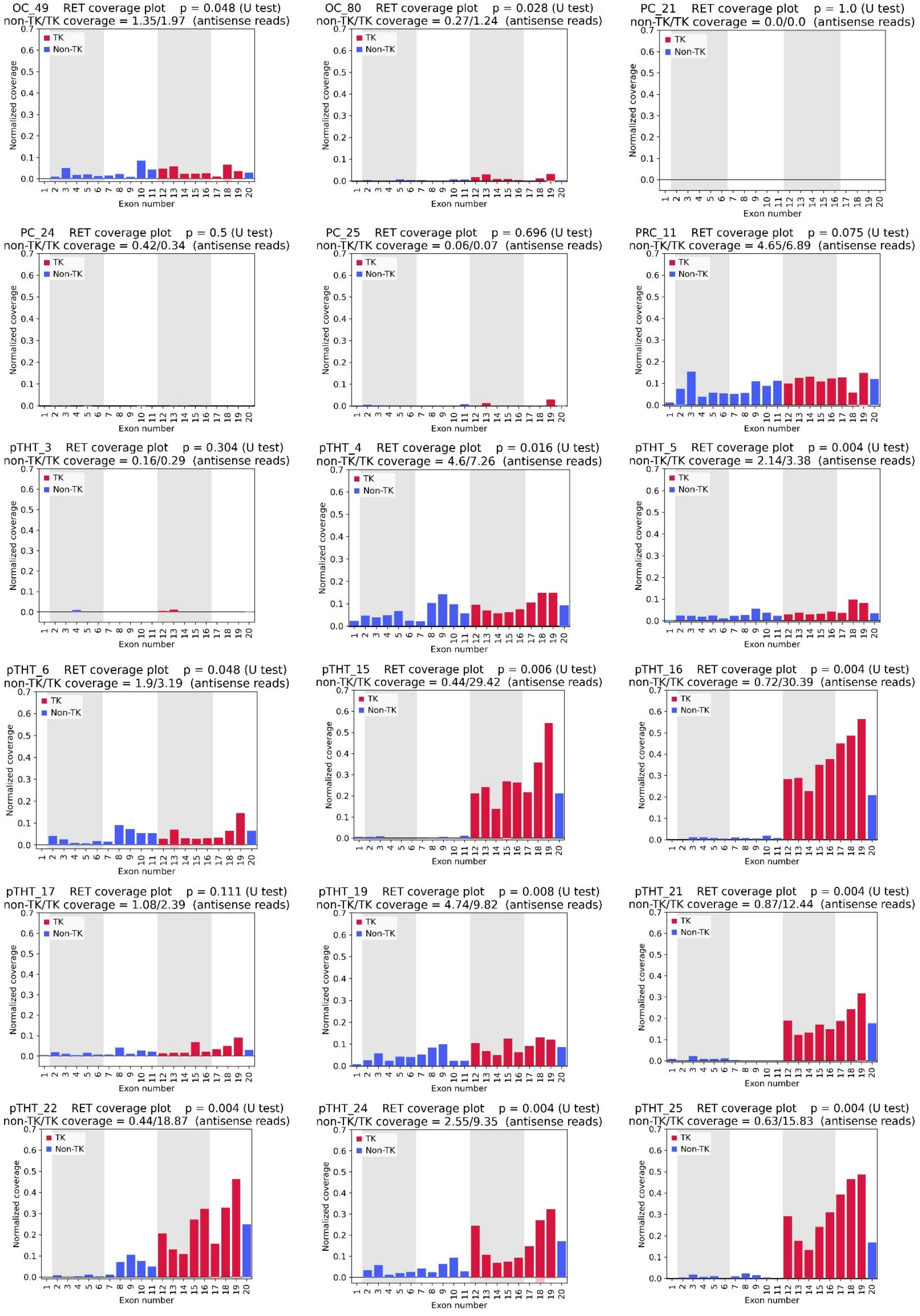
OC_49 RET coverage plot p = 0.048 (U test)
non-TK/TK coverage = 1.35/1.97 (antisense reads)
OC_80 RET coverage plot p = 0.028 (U test)
non-TK/TK coverage = 0.27/1.24 (antisense reads)
PC_21 RET coverage plot p = 1.0 (U test)
non-TK/TK coverage = 0.0/0.0 (antisense reads)
PC_24 RET coverage plot p = 0.5 (U test)
non-TK/TK coverage = 0.42/0.34 (antisense reads)
PC_25 RET coverage plot p = 0.696 (U test)
non-TK/TK coverage = 0.06/0.07 (antisense reads)
PRC_11 RET coverage plot p = 0.075 (U test)
non-TK/TK coverage = 4.65/6.89 (antisense reads)
pTHT_3 RET coverage plot p = 0.304 (U test)
non-TK/TK coverage = 0.16/0.29 (antisense reads)
pTHT_4 RET coverage plot p = 0.016 (U test)
non-TK/TK coverage = 4.6/7.26 (antisense reads)
pTHT_5 RET coverage plot p = 0.004 (U test)
non-TK/TK coverage = 2.14/3.38 (antisense reads)
pTHT_6 RET coverage plot p = 0.048 (U test)
non-TK/TK coverage = 1.9/3.19 (antisense reads)
pTHT_15 RET coverage plot p = 0.006 (U test)
non-TK/TK coverage = 0.44/29.42 (antisense reads)
pTHT_16 RET coverage plot p = 0.004 (U test)
non-TK/TK coverage = 0.72/30.39 (antisense reads)
pTHT_17 RET coverage plot p = 0.111 (U test)
non-TK/TK coverage = 1.08/2.39 (antisense reads)
pTHT_19 RET coverage plot p = 0.008 (U test)
non-TK/TK coverage = 4.74/9.82 (antisense reads)
pTHT_21 RET coverage plot p = 0.004 (U test)
non-TK/TK coverage = 0.87/12.44 (antisense reads)
pTHT_22 RET coverage plot p = 0.004 (U test)
non-TK/TK coverage = 0.44/18.87 (antisense reads)
pTHT_24 RET coverage plot p = 0.004 (U test)
non-TK/TK coverage = 2.55/9.35 (antisense reads)
pTHT_25 RET coverage plot p = 0.004 (U test)
non-TK/TK coverage = 0.63/15.83 (antisense reads)
TK
Non-TK
Normalized coverage
Exon number

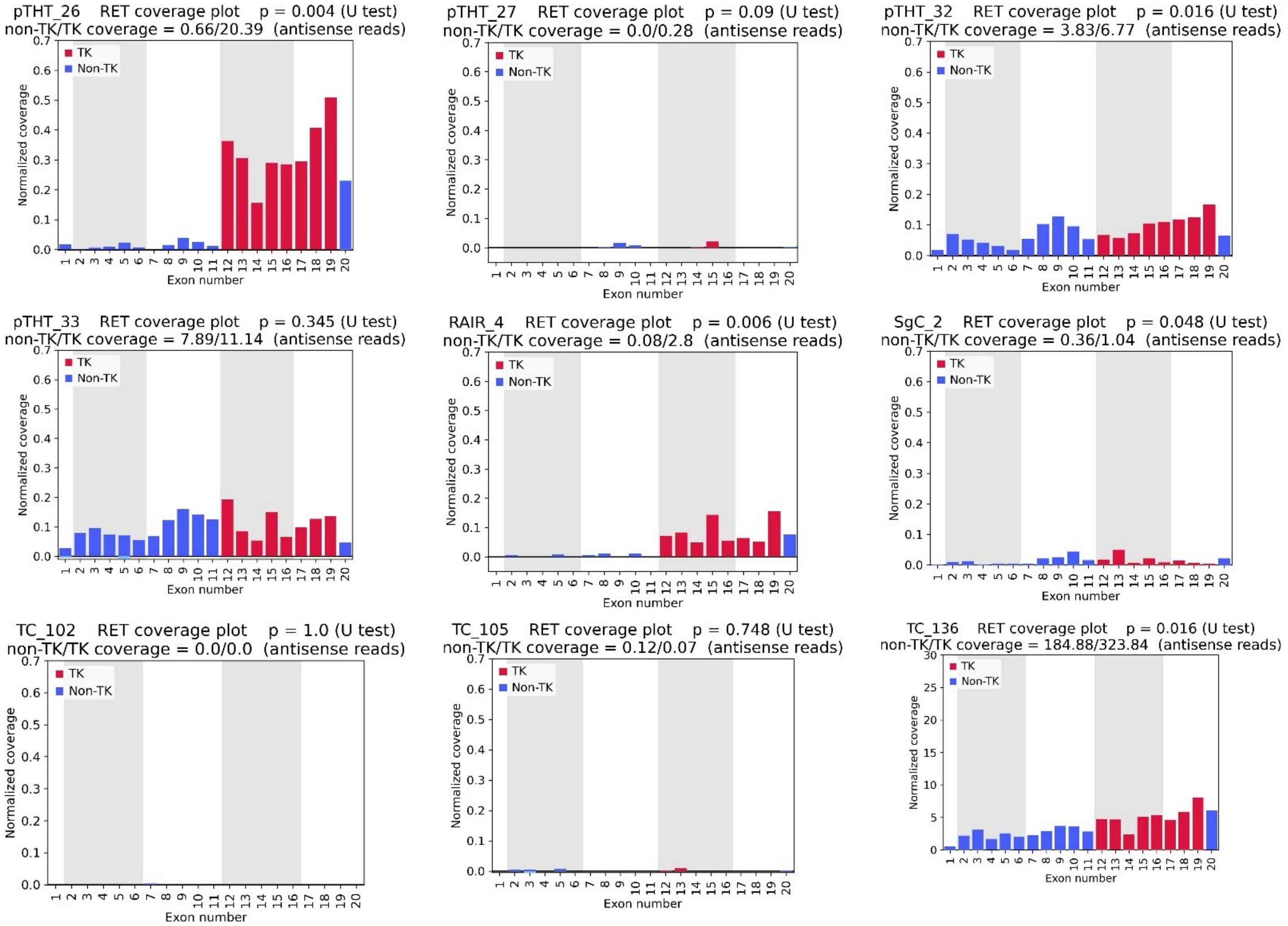

Figure S1. *RET* coverage plots based on RNA-seq data for samples from validation cohort, normalized on exon length and total read number in sample. TK – tyrosine kinase domain-related exons; non-TK – exons not related to the tyrosine kinase domain; non-TK/TK coverage – ratio of mean coverage of five non-TK exons (exons 2-6) and five TK exons (exons 12-16).

FGF_7

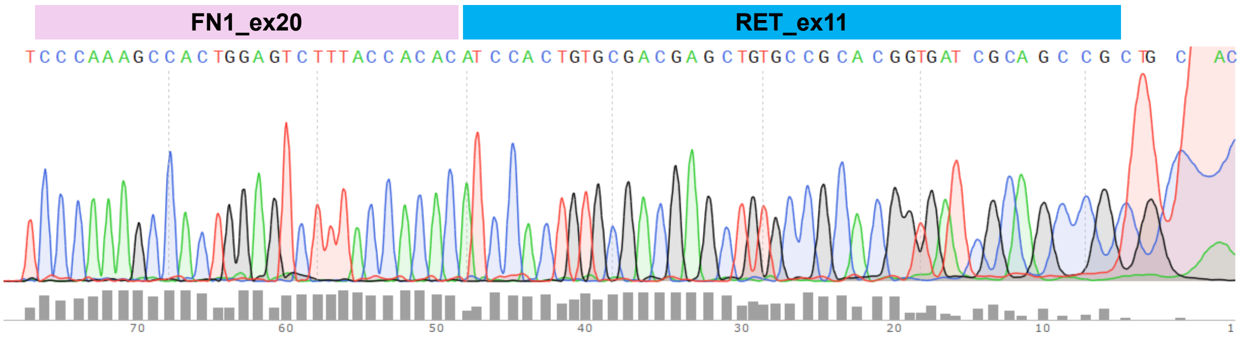

pTHT_15

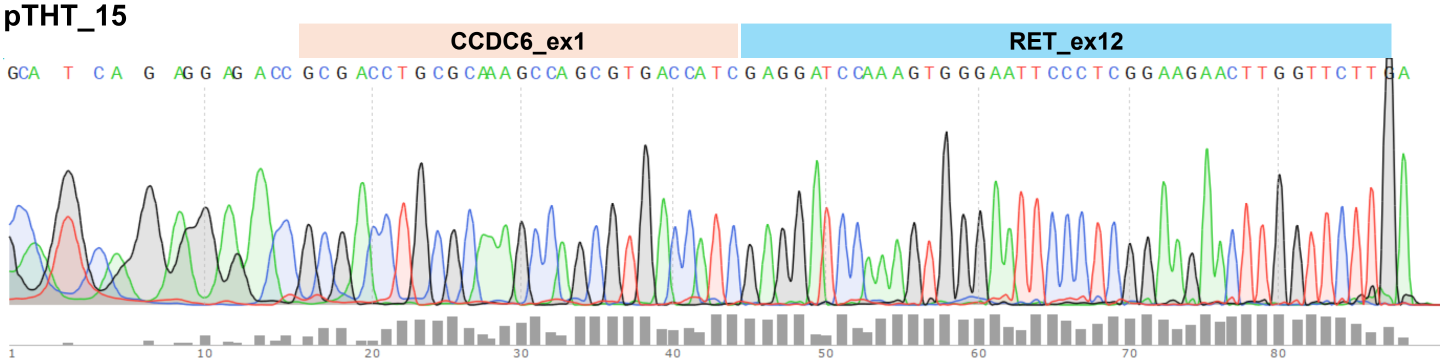

pTHT_16

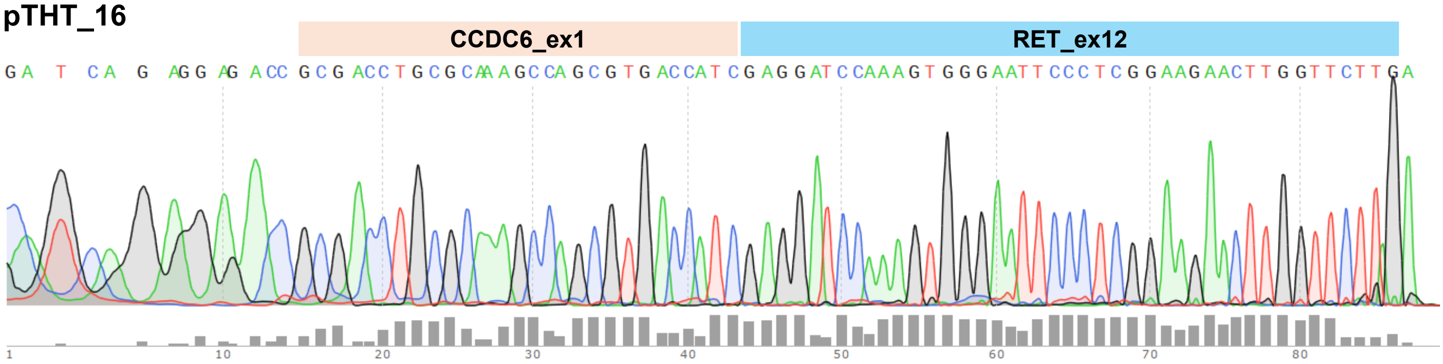

pTHT_21

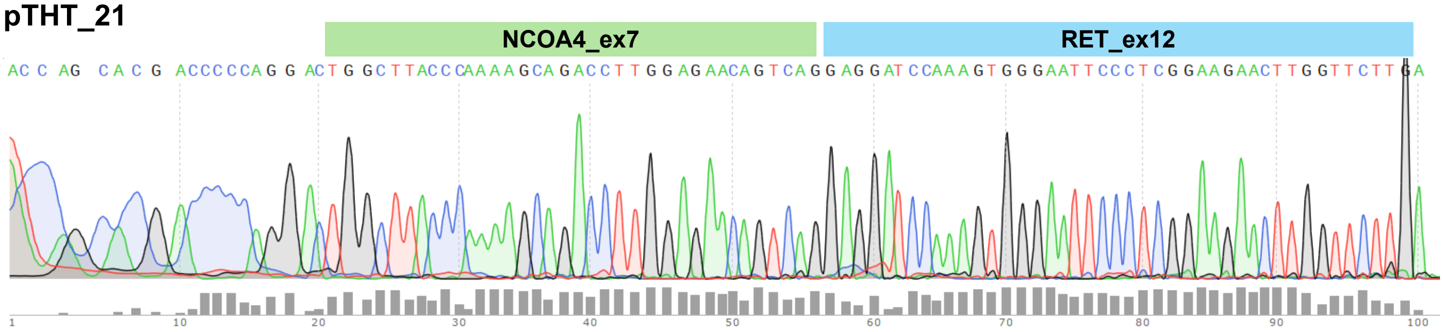

pTHT_22

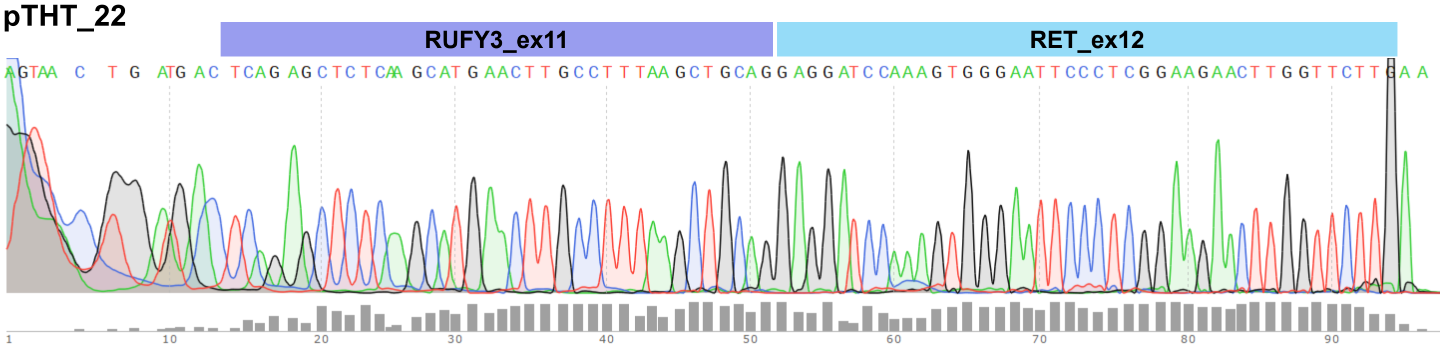

pTHT_24

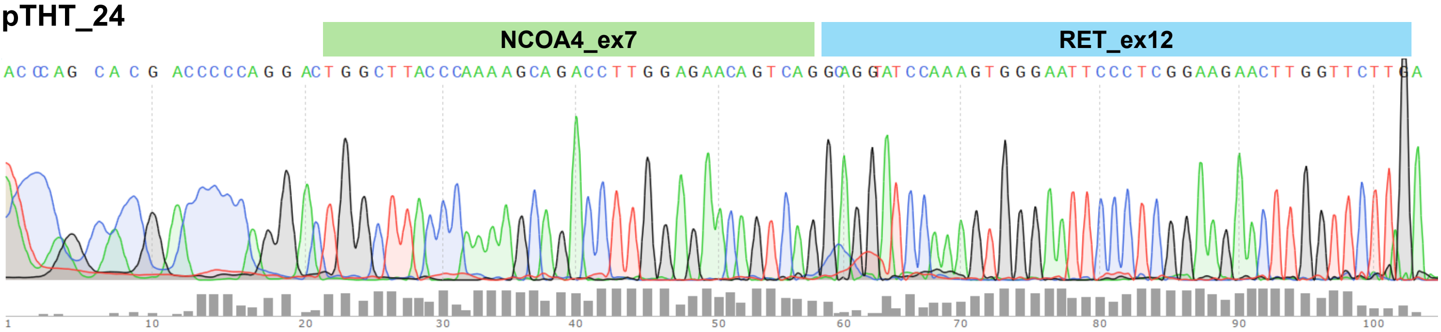

pTHT_25

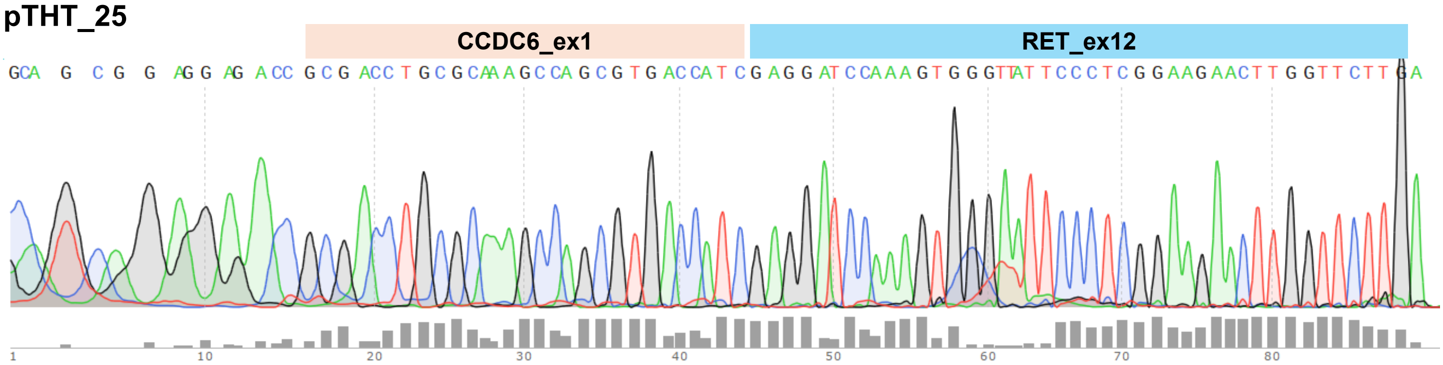

pTHT_26

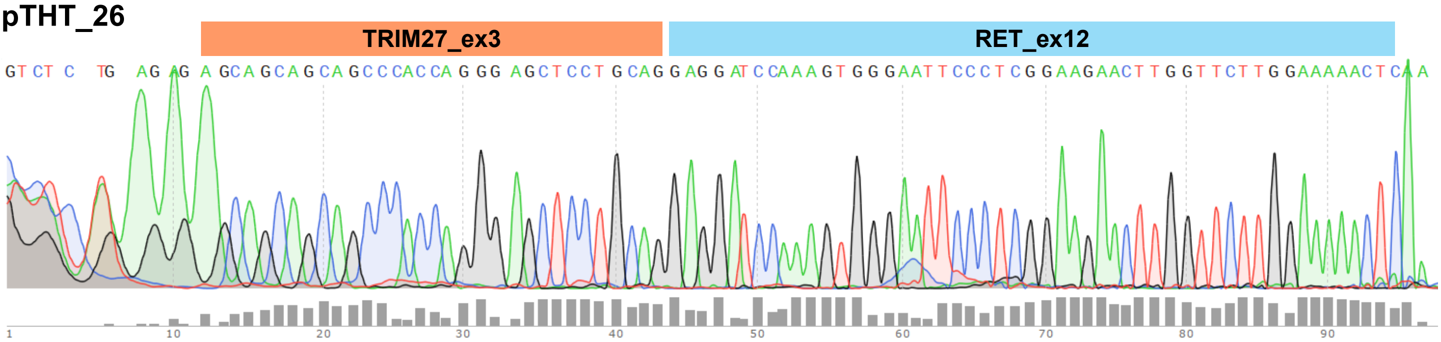

RAIR_4

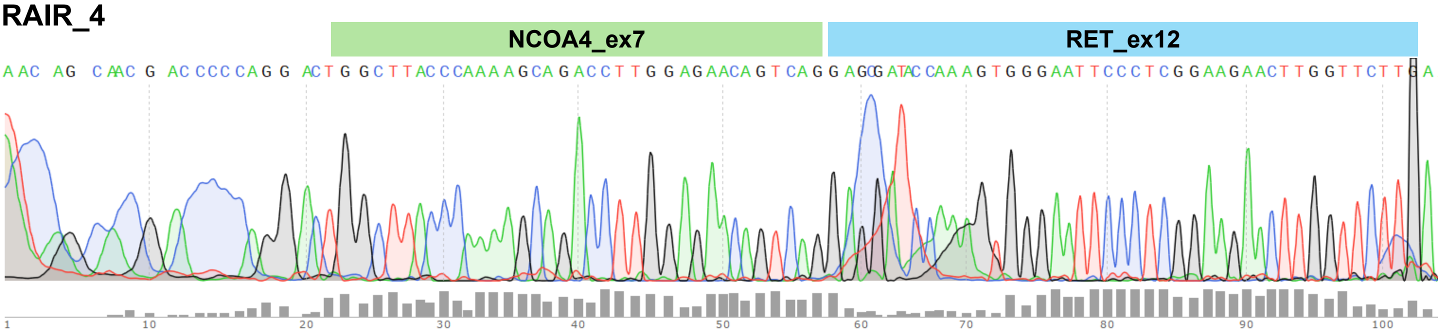

THT_51

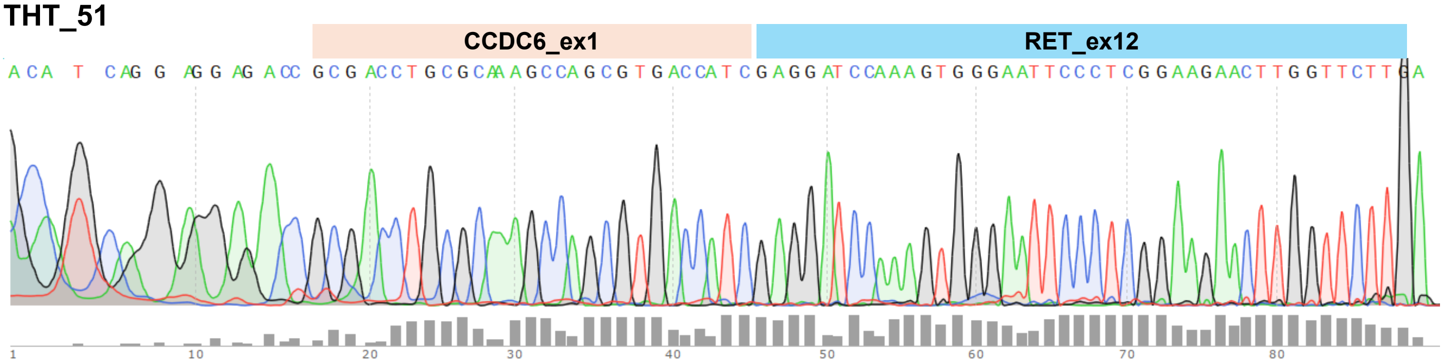

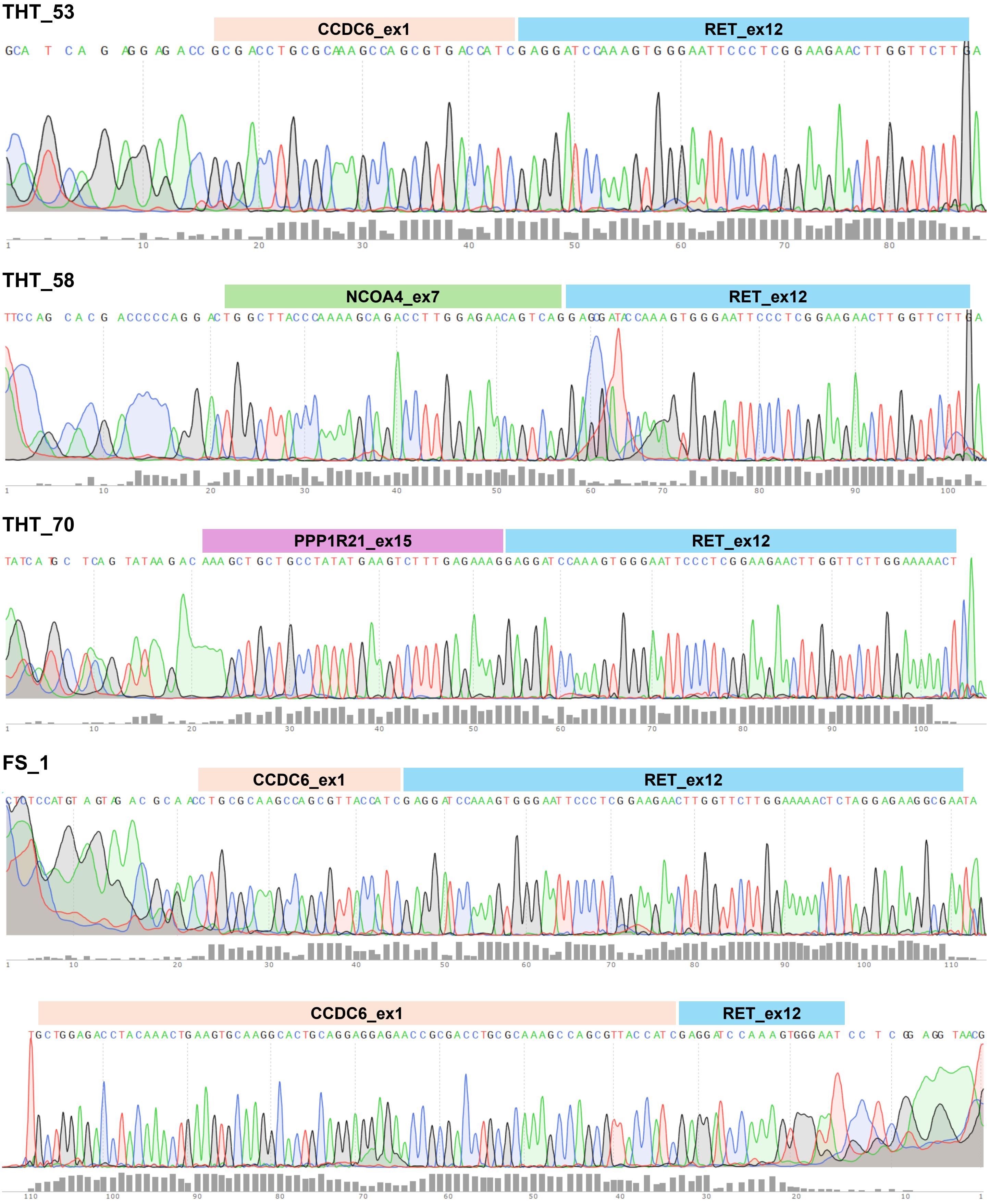

Figure S2. Results of *RET* fusion validation by Sanger sequencing.

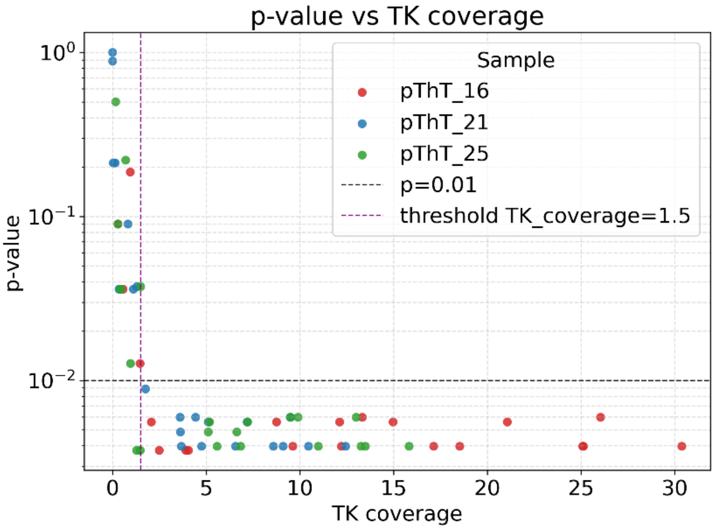

Figure S3. Dependence of statistical significance for *RET* coverage asymmetry on the coverage depth of TK-related exons 12–16. Values are shown for subsamples of three *RET* fusion-positive samples, created by randomly selecting varying numbers of reads from the original FASTQ files. The horizontal dashed line denotes the threshold *p*-value of 0.01. The vertical dashed line indicates the simulated minimum coverage depth threshold, below which the detection of *RET* coverage asymmetry in RNA-seq becomes unreliable. TK – tyrosine kinase domain-related exons 12-16.

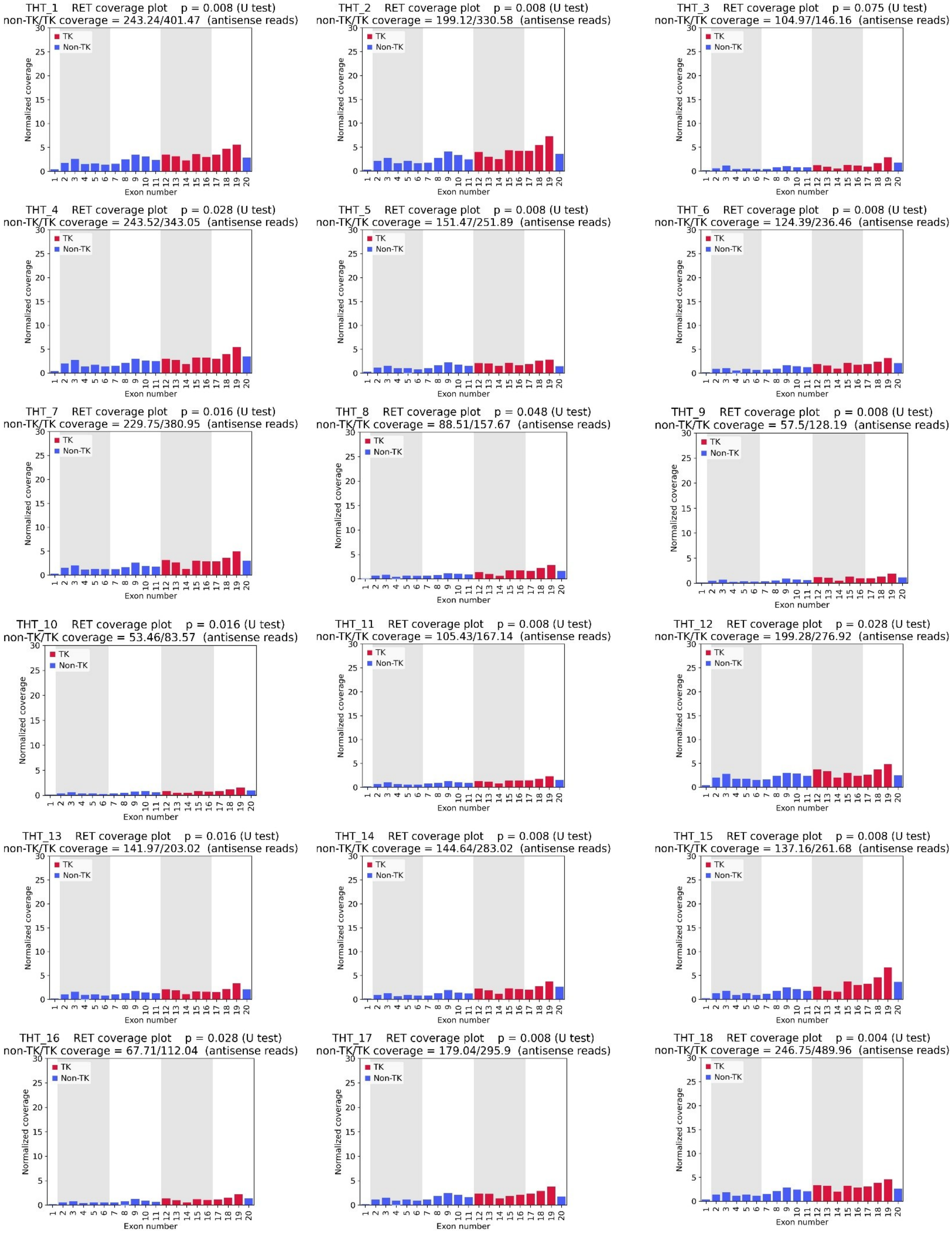
THT_1 RET coverage plot p = 0.008 (U test)
non-TK/TK coverage = 243.24/401.47 (antisense reads)
THT_2 RET coverage plot p = 0.008 (U test)
non-TK/TK coverage = 199.12/330.58 (antisense reads)
THT_3 RET coverage plot p = 0.075 (U test)
non-TK/TK coverage = 104.97/146.16 (antisense reads)
THT_4 RET coverage plot p = 0.028 (U test)
non-TK/TK coverage = 243.52/343.05 (antisense reads)
THT_5 RET coverage plot p = 0.008 (U test)
non-TK/TK coverage = 151.47/251.89 (antisense reads)
THT_6 RET coverage plot p = 0.008 (U test)
non-TK/TK coverage = 124.39/236.46 (antisense reads)
THT_7 RET coverage plot p = 0.016 (U test)
non-TK/TK coverage = 229.75/380.95 (antisense reads)
THT_8 RET coverage plot p = 0.048 (U test)
non-TK/TK coverage = 88.51/157.67 (antisense reads)
THT_9 RET coverage plot p = 0.008 (U test)
non-TK/TK coverage = 57.5/128.19 (antisense reads)
THT_10 RET coverage plot p = 0.016 (U test)
non-TK/TK coverage = 53.46/83.57 (antisense reads)
THT_11 RET coverage plot p = 0.008 (U test)
non-TK/TK coverage = 105.43/167.14 (antisense reads)
THT_12 RET coverage plot p = 0.028 (U test)
non-TK/TK coverage = 199.28/276.92 (antisense reads)
THT_13 RET coverage plot p = 0.016 (U test)
non-TK/TK coverage = 141.97/203.02 (antisense reads)
THT_14 RET coverage plot p = 0.008 (U test)
non-TK/TK coverage = 144.64/283.02 (antisense reads)
THT_15 RET coverage plot p = 0.008 (U test)
non-TK/TK coverage = 137.16/261.68 (antisense reads)
THT_16 RET coverage plot p = 0.028 (U test)
non-TK/TK coverage = 67.71/112.04 (antisense reads)
THT_17 RET coverage plot p = 0.008 (U test)
non-TK/TK coverage = 179.04/295.9 (antisense reads)
THT_18 RET coverage plot p = 0.004 (U test)
non-TK/TK coverage = 246.75/489.96 (antisense reads)
TK
Non-TK
Normalized coverage
Exon number

THT_19 RET coverage plot p = 0.008 (U test)
non-TK/TK coverage = 140.74/290.58 (antisense reads)

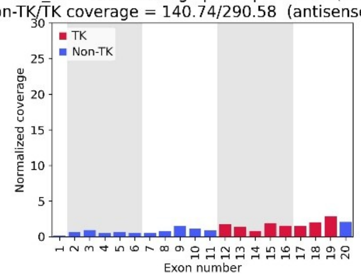

THT_20 RET coverage plot p = 0.008 (U test)
non-TK/TK coverage = 127.76/255.53 (antisense reads)

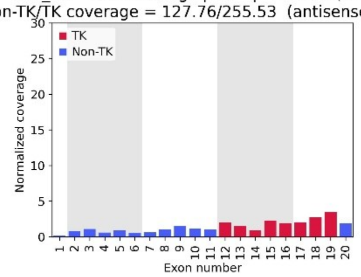

THT_21 RET coverage plot p = 0.008 (U test)
non-TK/TK coverage = 235.33/416.24 (antisense reads)

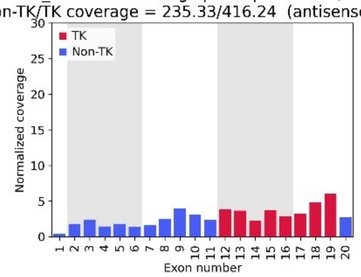

THT_22 RET coverage plot p = 0.008 (U test)
non-TK/TK coverage = 124.04/217.07 (antisense reads)

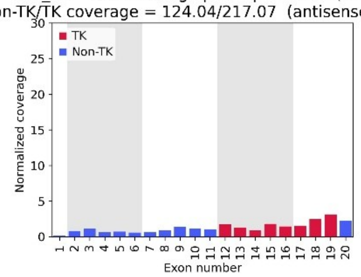

THT_23 RET coverage plot p = 0.008 (U test)
non-TK/TK coverage = 121.41/265.65 (antisense reads)

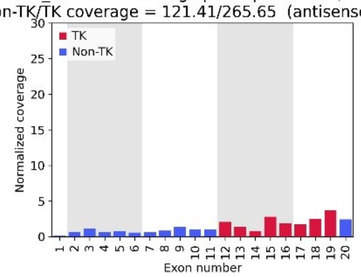

THT_24 RET coverage plot p = 0.008 (U test)
non-TK/TK coverage = 123.13/265.64 (antisense reads)

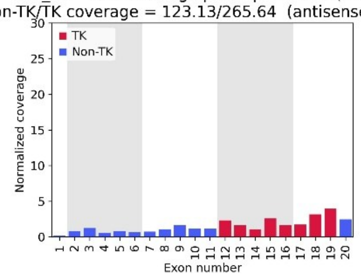

THT_25 RET coverage plot p = 0.016 (U test)
non-TK/TK coverage = 131.17/210.89 (antisense reads)

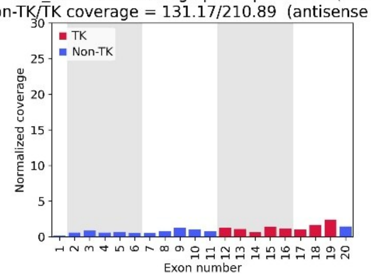

THT_26 RET coverage plot p = 0.028 (U test)
non-TK/TK coverage = 67.43/105.67 (antisense reads)

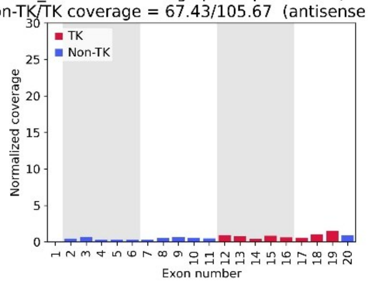

THT_27 RET coverage plot p = 0.004 (U test)
non-TK/TK coverage = 24.99/49.93 (antisense reads)

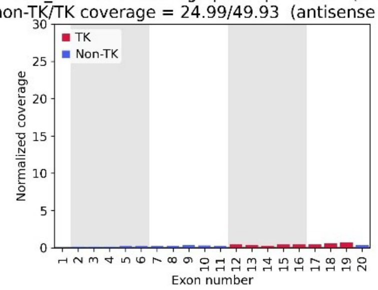

THT_28 RET coverage plot p = 0.016 (U test)
non-TK/TK coverage = 45.57/93.73 (antisense reads)

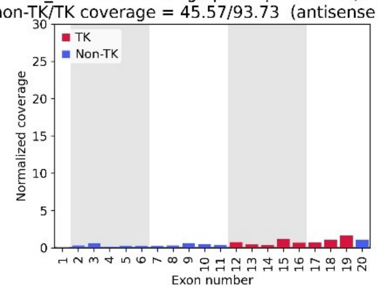

THT_29 RET coverage plot p = 0.008 (U test)
non-TK/TK coverage = 109.65/192.53 (antisense reads)

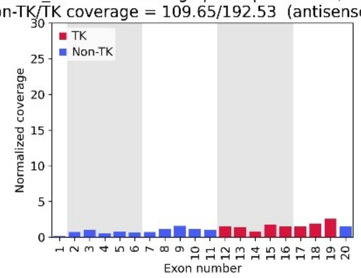

THT_30 RET coverage plot p = 0.008 (U test)
non-TK/TK coverage = 151.66/294.96 (antisense reads)

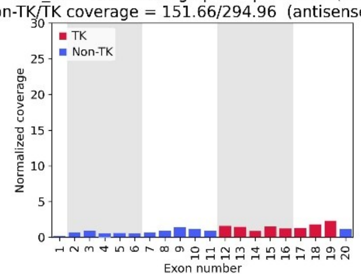

THT_31 RET coverage plot p = 0.004 (U test)
non-TK/TK coverage = 176.14/305.91 (antisense reads)

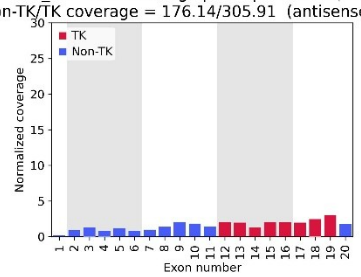

THT_32 RET coverage plot p = 0.008 (U test)
non-TK/TK coverage = 127.79/222.68 (antisense reads)

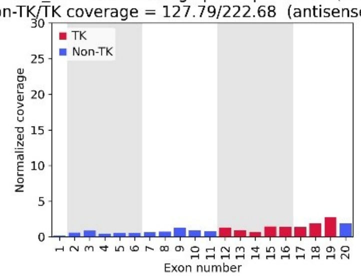

THT_33 RET coverage plot p = 0.008 (U test)
non-TK/TK coverage = 138.56/253.53 (antisense reads)

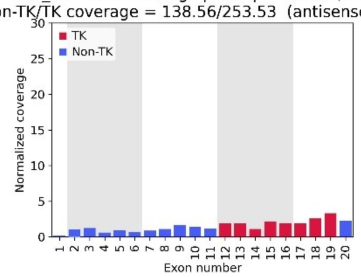

THT_34 RET coverage plot p = 0.008 (U test)
non-TK/TK coverage = 65.44/136.16 (antisense reads)

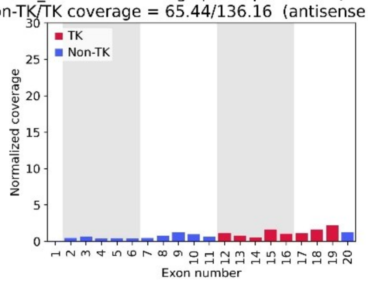

THT_35 RET coverage plot p = 0.008 (U test)
non-TK/TK coverage = 172.97/317.16 (antisense reads)

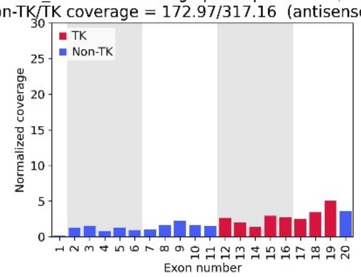

THT_36 RET coverage plot p = 0.008 (U test)
non-TK/TK coverage = 188.46/303.12 (antisense reads)

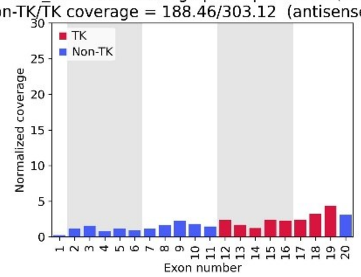

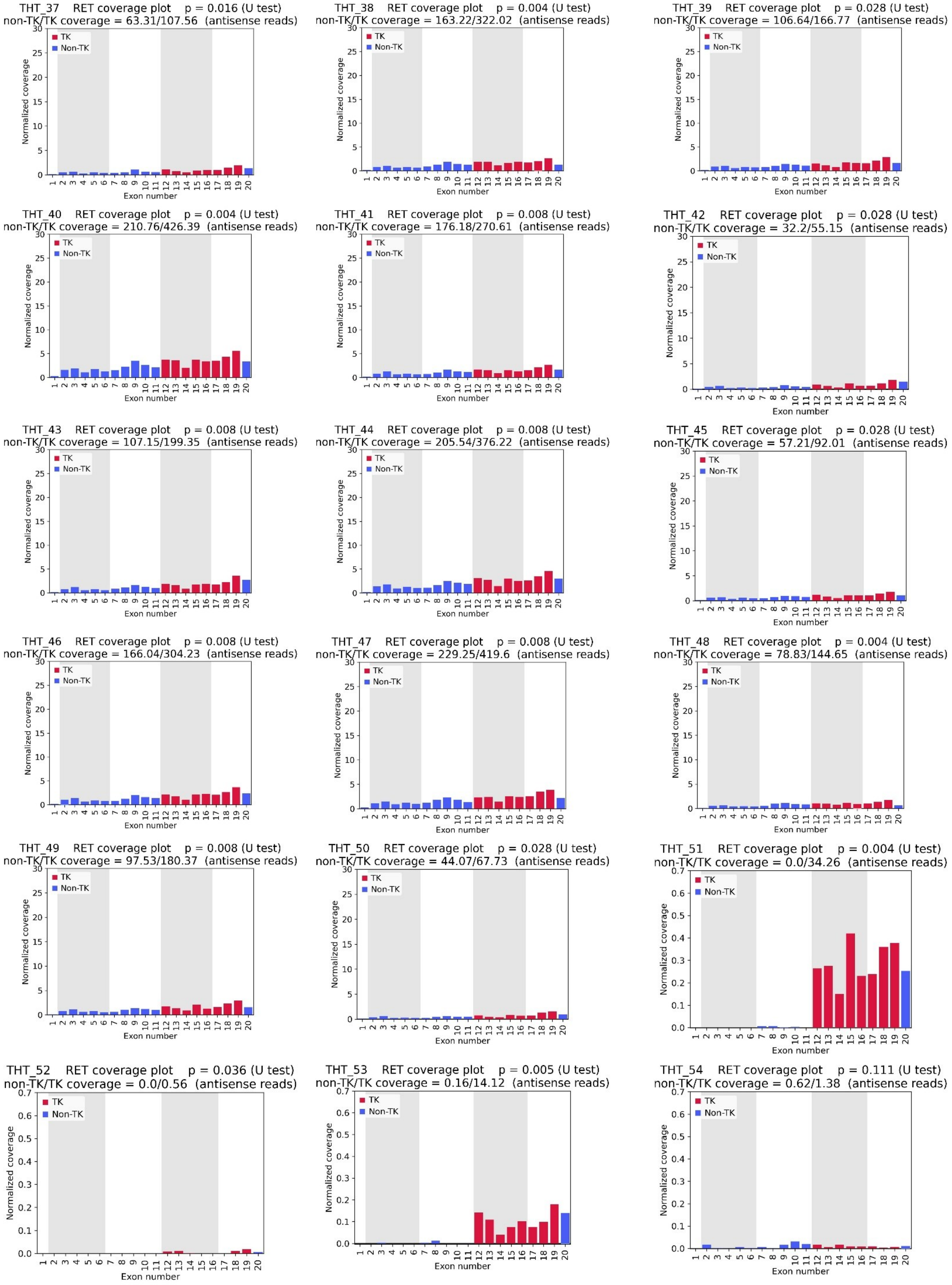
THT_37 RET coverage plot p = 0.016 (U test)
non-TK/TK coverage = 63.31/107.56 (antisense reads)
THT_38 RET coverage plot p = 0.004 (U test)
non-TK/TK coverage = 163.22/322.02 (antisense reads)
THT_39 RET coverage plot p = 0.028 (U test)
non-TK/TK coverage = 106.64/166.77 (antisense reads)
THT_40 RET coverage plot p = 0.004 (U test)
non-TK/TK coverage = 210.76/426.39 (antisense reads)
THT_41 RET coverage plot p = 0.008 (U test)
non-TK/TK coverage = 176.18/270.61 (antisense reads)
THT_42 RET coverage plot p = 0.028 (U test)
non-TK/TK coverage = 32.2/55.15 (antisense reads)
THT_43 RET coverage plot p = 0.008 (U test)
non-TK/TK coverage = 107.15/199.35 (antisense reads)
THT_44 RET coverage plot p = 0.008 (U test)
non-TK/TK coverage = 205.54/376.22 (antisense reads)
THT_45 RET coverage plot p = 0.028 (U test)
non-TK/TK coverage = 57.21/92.01 (antisense reads)
THT_46 RET coverage plot p = 0.008 (U test)
non-TK/TK coverage = 166.04/304.23 (antisense reads)
THT_47 RET coverage plot p = 0.008 (U test)
non-TK/TK coverage = 229.25/419.6 (antisense reads)
THT_48 RET coverage plot p = 0.004 (U test)
non-TK/TK coverage = 78.83/144.65 (antisense reads)
THT_49 RET coverage plot p = 0.008 (U test)
non-TK/TK coverage = 97.53/180.37 (antisense reads)
THT_50 RET coverage plot p = 0.028 (U test)
non-TK/TK coverage = 44.07/67.73 (antisense reads)
THT_51 RET coverage plot p = 0.004 (U test)
non-TK/TK coverage = 0.0/34.26 (antisense reads)
THT_52 RET coverage plot p = 0.036 (U test)
non-TK/TK coverage = 0.0/0.56 (antisense reads)
THT_53 RET coverage plot p = 0.005 (U test)
non-TK/TK coverage = 0.16/14.12 (antisense reads)
THT_54 RET coverage plot p = 0.111 (U test)
non-TK/TK coverage = 0.62/1.38 (antisense reads)
TK
Non-TK
Normalized coverage
Exon number

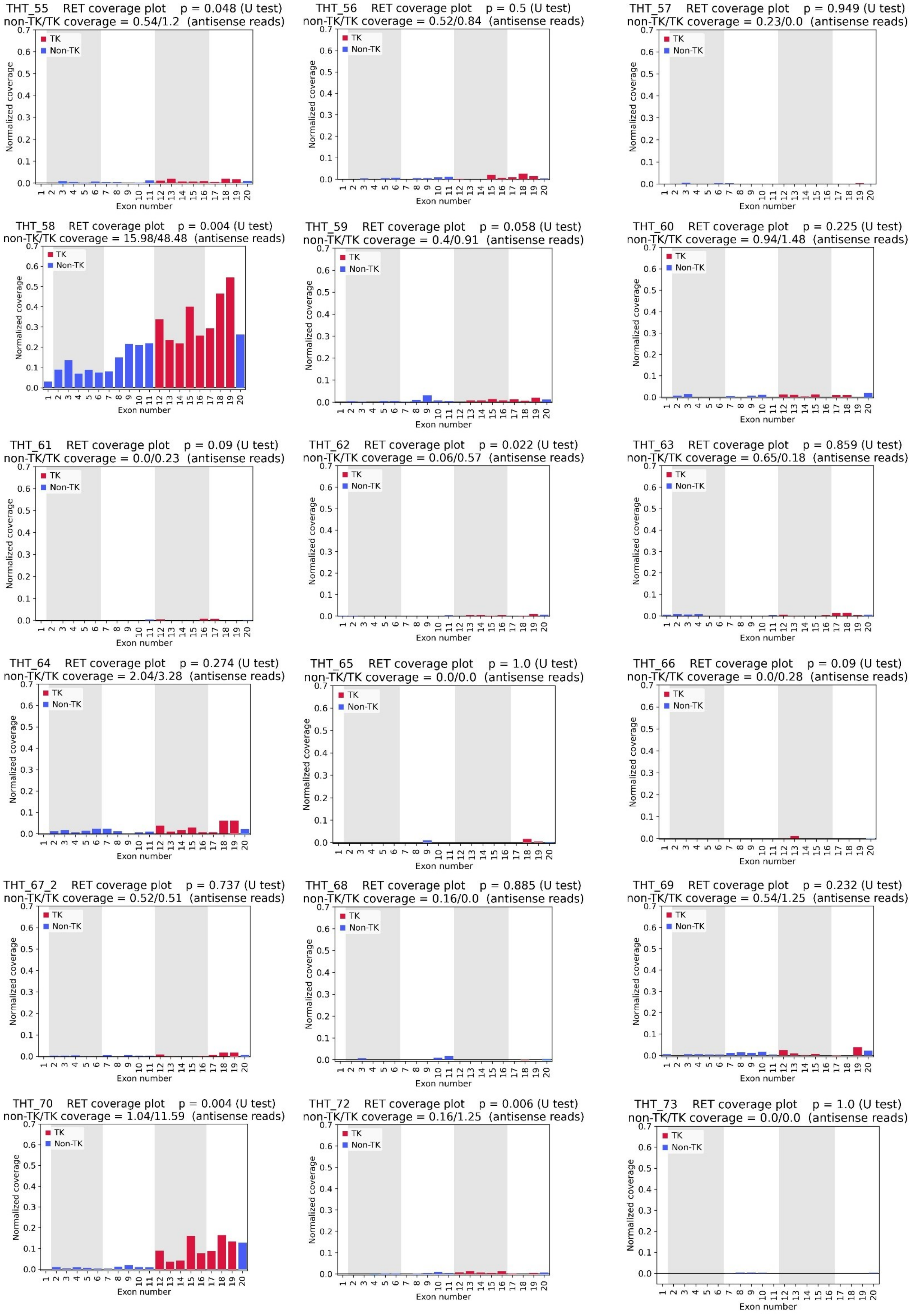
THT_55 RET coverage plot p = 0.048 (U test)
non-TK/TK coverage = 0.54/1.2 (antisense reads)
THT_56 RET coverage plot p = 0.5 (U test)
non-TK/TK coverage = 0.52/0.84 (antisense reads)
THT_57 RET coverage plot p = 0.949 (U test)
non-TK/TK coverage = 0.23/0.0 (antisense reads)
THT_58 RET coverage plot p = 0.004 (U test)
non-TK/TK coverage = 15.98/48.48 (antisense reads)
THT_59 RET coverage plot p = 0.058 (U test)
non-TK/TK coverage = 0.4/0.91 (antisense reads)
THT_60 RET coverage plot p = 0.225 (U test)
non-TK/TK coverage = 0.94/1.48 (antisense reads)
THT_61 RET coverage plot p = 0.09 (U test)
non-TK/TK coverage = 0.0/0.23 (antisense reads)
THT_62 RET coverage plot p = 0.022 (U test)
non-TK/TK coverage = 0.06/0.57 (antisense reads)
THT_63 RET coverage plot p = 0.859 (U test)
non-TK/TK coverage = 0.65/0.18 (antisense reads)
THT_64 RET coverage plot p = 0.274 (U test)
non-TK/TK coverage = 2.04/3.28 (antisense reads)
THT_65 RET coverage plot p = 1.0 (U test)
non-TK/TK coverage = 0.0/0.0 (antisense reads)
THT_66 RET coverage plot p = 0.09 (U test)
non-TK/TK coverage = 0.0/0.28 (antisense reads)
THT_67_2 RET coverage plot p = 0.737 (U test)
non-TK/TK coverage = 0.52/0.51 (antisense reads)
THT_68 RET coverage plot p = 0.885 (U test)
non-TK/TK coverage = 0.16/0.0 (antisense reads)
THT_69 RET coverage plot p = 0.232 (U test)
non-TK/TK coverage = 0.54/1.25 (antisense reads)
THT_70 RET coverage plot p = 0.004 (U test)
non-TK/TK coverage = 1.04/11.59 (antisense reads)
THT_72 RET coverage plot p = 0.006 (U test)
non-TK/TK coverage = 0.16/1.25 (antisense reads)
THT_73 RET coverage plot p = 1.0 (U test)
non-TK/TK coverage = 0.0/0.0 (antisense reads)
TK
Non-TK
Normalized coverage
Exon number

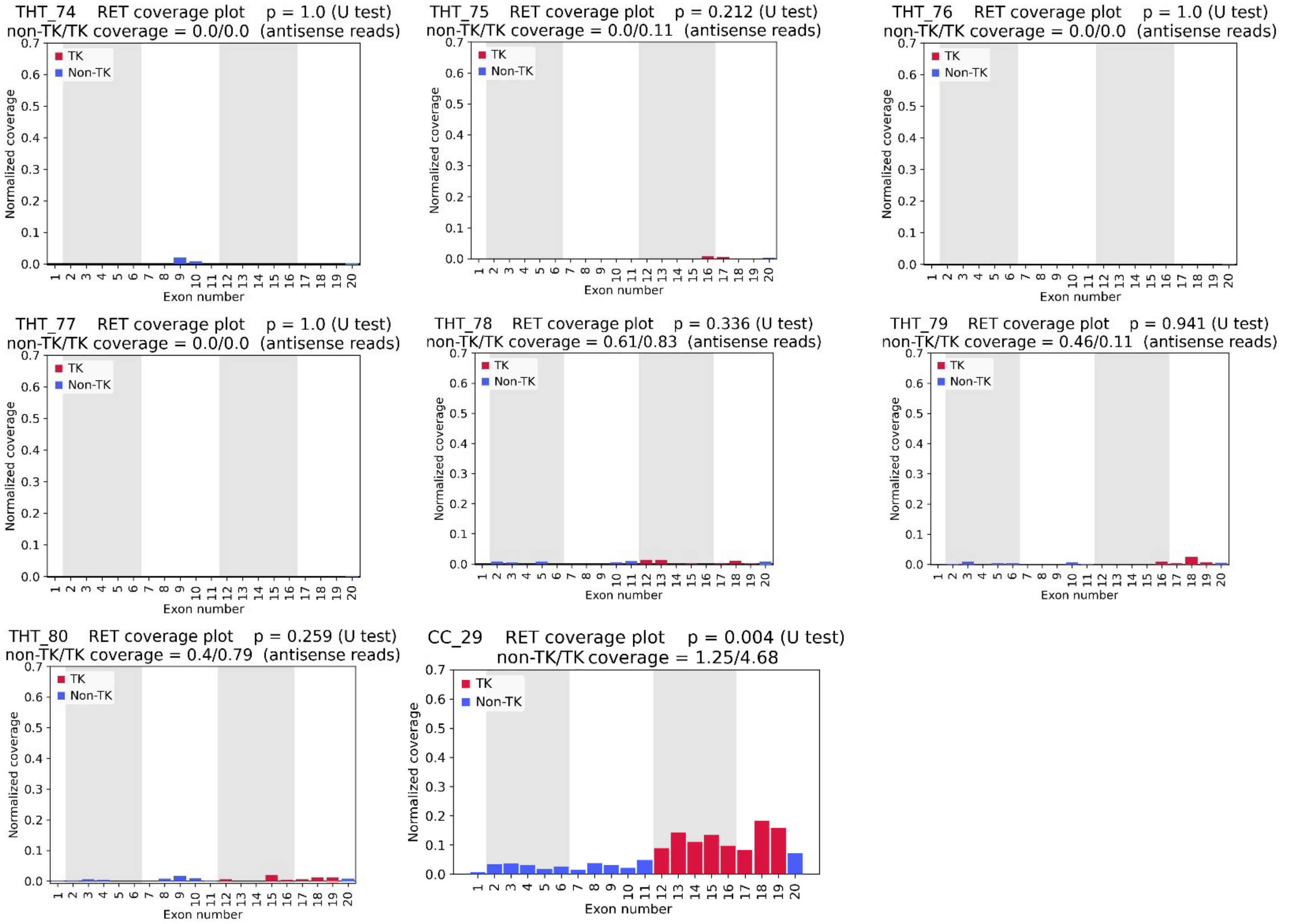

Figure S4. *RET* coverage plots based on RNA-seq data for samples from the additional validation cohort of thyroid cancer samples. Coverage is, normalized on exon length and total read number in sample. TK – tyrosine kinase domain-related exons; non-TK – exons not related to the tyrosine kinase domain; non-TK/TK coverage – ratio of mean coverage of five non-TK exons (exons 2-6) and five TK exons (exons 12-16).

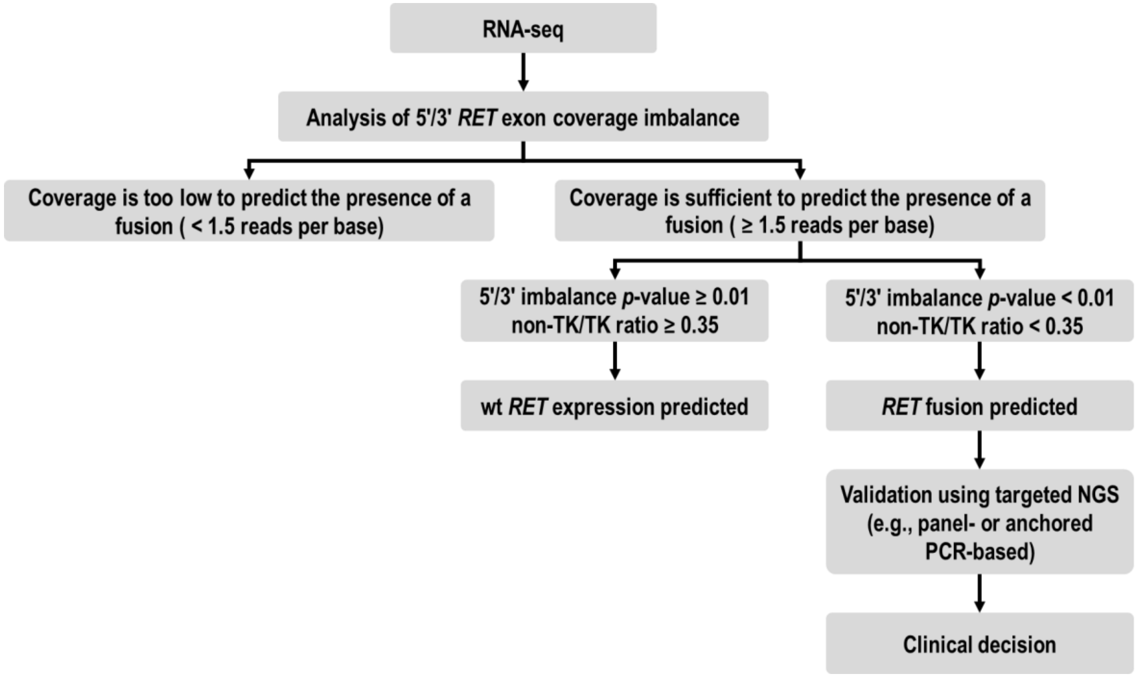

Figure S5. Proposed workflow for *RET* fusion detection using RNA-seq coverage imbalance analysis.
